# Facet‐Engineered (100)‐Oriented MoO_2_ Nanoribbons for Broadband Self‐Powered Photodetection

**DOI:** 10.1002/advs.202510753

**Published:** 2025-08-25

**Authors:** Haojian Lin, Ximiao Wang, Tianrong Yi, Jidong Liu, Jiahao Wu, Shaojing Liu, Yang Chai, Fei Liu, Di Wu, Huanjun Chen, Wenjing Zhang

**Affiliations:** ^1^ State Key Laboratory of Radio Frequency Heterogeneous Integration International Collaborative Laboratory of 2D Materials for Optoelectronics Science and Technology of the Ministry of Education Institute of Microscale Optoelectronics Shenzhen University Shenzhen 518060 China; ^2^ State Key Laboratory of Optoelectronic Materials and Technologies Guangdong Province Key Laboratory of Display Material and Technology School of Electronics and Information Technology Sun Yat‐sen University Guangzhou 510275 China; ^3^ College of Electronic Engineering Huainan Normal University Huainan 232038 China; ^4^ School of Electronic and Computer Engineering Peking University Shenzhen Graduate School Shenzhen 518055 China; ^5^ Department of Applied Physics The Hong Kong Polytechnic University Kowloon Hong Kong 999077 China

**Keywords:** atmospheric pressure chemical vapor deposition method, broadband photodetectors, flexible photodetectors, MoO2, nanoribbon, self‐powered photodetection

## Abstract

Broadband photodetection plays a vital role in aerospace applications, biomedical imaging, and advanced communication systems. While molybdenum dioxide (MoO_2_) exhibits exceptional electrical conductivity, carrier mobility, and environmental stability, its potential for photodetection has remained unrealized, with existing literature reporting negligible optoelectronic responses. Here, we unlock latent photoresponsivity of MoO_2_ by facet engineering, demonstrating that exposing the (100) crystallographic plane activates its intrinsic photoelectric conversion. Using atmospheric‐pressure chemical vapor deposition, we successfully fabricated large‐area arrays of (100)‐oriented MoO_2_ nanoribbons. The resulting flexible photodetector on polyethylene glycol terephthalate (PET) substrate exhibits unprecedented performance, achieving broadband detection from visible to long‐wave infrared (LWIR: 0.5–10.5 µm) range without external bias. The device demonstrates a fivefold enhancement in responsivity compared to rigid substrate configurations, reaching 107.31 mA W^−1^ at 10.5 µm wavelength with an exceptionally low noise‐equivalent power (*NEP*) of 6.64 pW Hz^−0.5^, surpassing all self‐powered photodetectors reported to date. Comprehensive characterization reveals distinct photoresponse mechanisms: photothermoelectric effects dominate on silicon substrates, while photobolometric behavior prevails in flexible configurations. These findings not only resolve the previously observed photoresponse limitations in MoO_2_ but also establish facet engineering as a general approach for developing high‐performance photodetectors based on metallic oxides, with significant implications for flexible optoelectronic applications.

## Graphical abstract

Facet engineering unlocks the latent broadband photoresponse of metallic MoO_2_ by exposing its (100) plane. Flexible photodetectors based on atmospheric‐pressure chemical vapor deposition‐grown nanoribbons achieve unprecedented self‐powered detection from visible to long‐wave infrared (0.5–10.5 µm) with 107.31 mA W^−1^ responsivity and 6.64 pW Hz^−0.5^ noise at 10.5 µm wavelength. Substrate‐dependent mechanisms—photothermoelectric versus photobolometric—resolve prior limitations, advancing flexible optoelectronics via facet‐tailored metallic oxides.

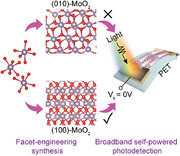

## 1 Introduction

Ultrabroadband photodetection has become increasingly vital for advanced applications such as high‐speed optical communication,^[^ ^1^ ^]^ precision spectroscopy,^[^ ^2^ ^]^ and real‐time environmental monitoring,^[^ ^3^ ^]^ owing to its ability to capture a wide spectral range with high resolution and rapid response. However, conventional photodetectors based on thin‐film semiconductors are often constrained by their inherent bandgap limitations, low carrier mobility, or inefficient optoelectronic conversion, restricting their performance to narrow spectral windows. To achieve true broadband detection, materials capable of efficient photon‐to‐electron conversion across a wide wavelength range are essential, yet the available options remain limited, and their synthesis is often complex and impractical for scalable deployment.^[^ ^4^ ^]^

Traditional narrow‐bandgap semiconductors like Hg_1‐x_Cd_x_Te and InGaAs have dominated photodetection in the visible to mid‐infrared range,^[^ ^4^ ^]^ but their performance declines at longer wavelengths, often requiring cryogenic cooling to suppress thermal noise. Quantum well structures,^[^ ^5^ ^]^ which rely on intraband transitions, offer an alternative but suffer from high noise at room temperature and are largely ineffective in the UV and visible regimes. Researchers have explored emerging materials such as 1D nanomaterials,^[^ ^6^, ^7^ ^]^ 2D van der Waals crystals,^[^ ^8^ ^]^ and hybrid heterostructures to overcome these limitations. For instance, graphene‐based detectors exhibit broadband operation but are hindered by low absorption and scalability challenges^[^ ^9^, ^10^, ^11^ ^]^ while perovskite materials like CH_3_NH_3_PbI_3_ show promise but degrade under ambient conditions.^[^ ^12^ ^]^

Among the emerging candidates, molybdenum dioxide (MoO_2_) stands out due to its metallic conductivity, zero bandgap characteristics,^[^ ^13^ ^]^ ultrahigh carrier mobility (≈5500 cm^2^ (V·s)^−1^),^[^ ^14^ ^]^ and robust chemical stability,^[^ ^15^, ^16^ ^]^ making it a promising material for ultrabroadband photodetection. While most synthesized MoO_2_ nanostructures adopt the (010) orientation and exhibit high electrical conductivity (≈10^5^ S m^−1^),^[^ ^17^, ^18^ ^]^ their optical absorption remains below 1%, severely limiting their photoresponse (Figure S1, Supporting Information). Theoretical studies suggest that (100)‐oriented MoO_2_ could achieve over 20% absorption across a broad spectral range (0.5–16 µm) while maintaining a high room‐temperature conductivity up to 10^8^ S m^−1^ (Figure S1, Supporting Information), but its synthesis remains challenging due to narrow growth windows and harsh conditions. To date, only low‐yield, impurity‐prone (100)‐oriented MoO_2_ nanostructures have been reported,^[^ ^18^ ^]^ leaving their optoelectronic properties largely unexplored and hindering progress in ultrabroadband photodetection applications.

In this work, we demonstrate the successful synthesis of large‐area (2 cm × 2 cm) metallic (100)‐oriented MoO_2_ nanoribbon arrays on *c*‐sapphire substrates via the atmospheric pressure chemical vapor deposition (APCVD) method. The (100) facet exposure plays a pivotal role in enabling exceptional optoelectronic properties, as these nanoribbons exhibit strong broadband light absorption exceeding 20% across the visible to infrared spectral range (0.5–10.5 µm). When fabricated into a self‐powered photodetector on a flexible polyethylene glycol terephthalate (PET) substrate, the (100)‐oriented MoO_2_ device achieves excellent performance metrics, including an ultralow *NEP* of 6.64 pW Hz^−0.5^ and a fast response time of ≈100 µs, both measured at 10.5 µm. These values surpass all self‐powered photodetectors, highlighting the advantage of the (100) facet in balancing high photoconductivity with minimal electronic noise. To elucidate the underlying physical mechanisms, we combine first‐principle calculations, COMSOL simulations, and spatially resolved photocurrent mapping. This multimodal approach reveals that the zero‐bias photoresponse originates from the (100) facet‐dominated efficient free carrier absorption. The (100) surface orientation not only enhances light–matter interaction but also facilitates strong carrier absorption, providing a new design paradigm for high‐performance, broadband photodetection without external power requirements.

## 2 Results and Discussion

### 2.1 Synthesis and Structure Characterization of Metallic (100)‐Oriented MoO_2_ Nanoribbon Arrays

**Figure 1:**
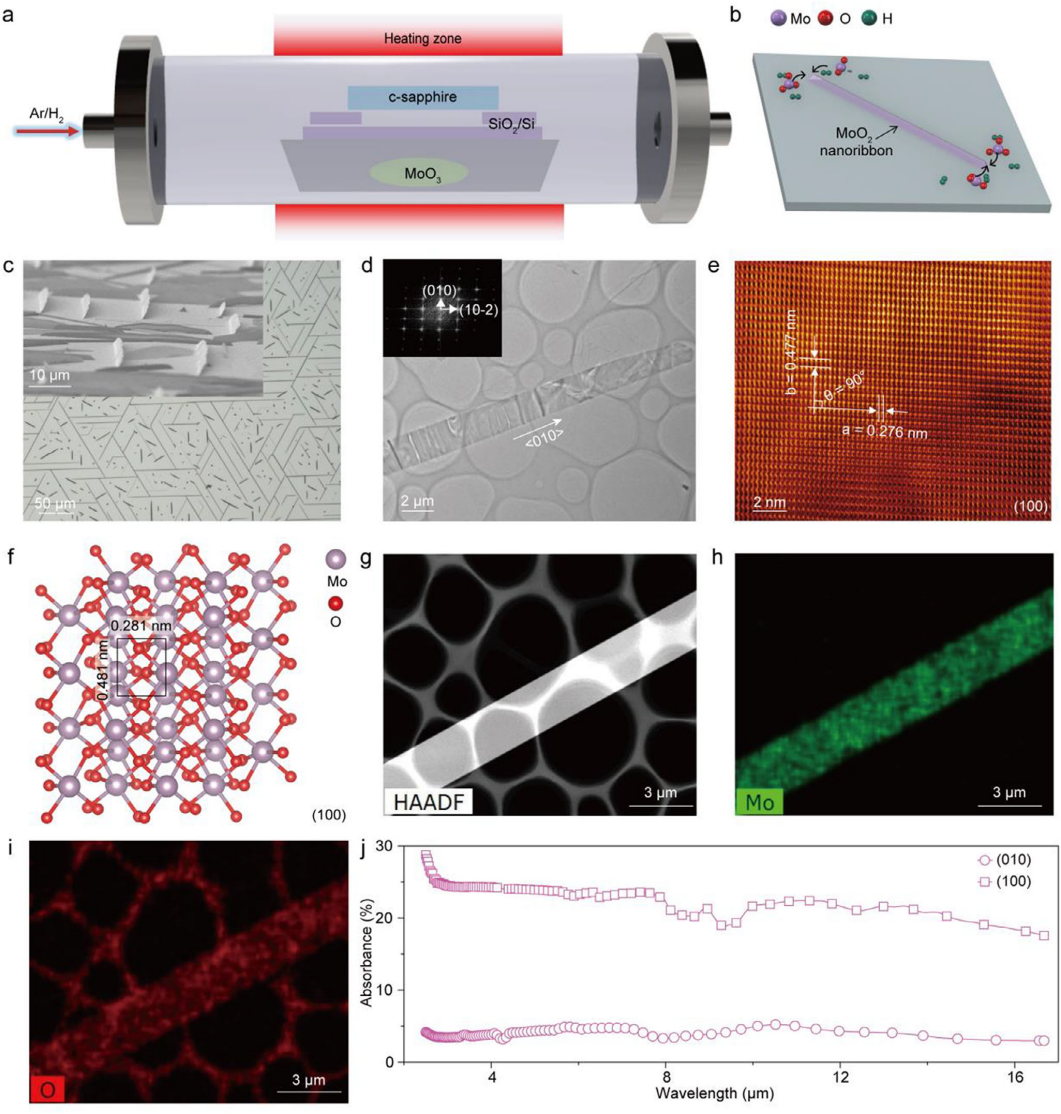
Synthesis and characterization of (100)‐oriented MoO_2_ nanoribbons. a) Schematic diagram of the APCVD system for growing (100)‐oriented MoO_2_ nanoribbons. b) The formation mechanism of (100)‐oriented MoO_2_ nanoribbon. c) Optical microscope image of the (100)‐oriented MoO_2_ nanoribbons on *c*‐sapphire, and the inset gives their high‐solution cross‐section image. d,e) Typical TEM and HRTEM images of a (100)‐oriented MoO_2_ nanoribbon. And the corresponding FFT pattern is shown in the inset. f) Top view of the rectangular unit cell of the (100)‐oriented MoO_2_ nanoribbon by DFT calculation. g–i) HAADF‐STEM and EDX mapping images of a MoO_2_ nanoribbon. j) Experimental light absorbance spectra of the (100)‐ and (010)‐oriented MoO_2_ nanostructures in a wide spectral range from 3 to 16 µm, respectively.

**Figure** **1**a illustrates the APCVD system developed by our group for the synthesis of MoO_2_ nanoribbons. In this setup, MoO_3_ powders are placed in a quartz boat as the precursor, and a (0001)‐oriented *c*‐plane sapphire wafer is positioned above the source as the growth substrate. The proposed growth mechanism for (100)‐oriented MoO_2_ nanoribbons is depicted in Figure 1b. Upon heating, MoO_3_ sublimes and is subsequently reduced to MoO_2_ vapor by H_2_ gas. These MoO_2_ species nucleate as initial seed crystals on the *c*‐sapphire surface. At the early stage of growth, due to the low local concentration of MoO_2_, subsequent adatoms preferentially adsorb onto the (010) facets of the seeds—those with the highest surface Gibbs free energy—following a surface‐confined successive growth model.^[^ ^18^, ^19^ ^]^ As growth proceeds, the MoO_2_ nanocrystals extend along the (100) direction, which possesses the lowest surface Gibbs free energy, thus driving the anisotropic elongation into nanoribbons. Distinct from traditional chalcogen‐assisted methods, our approach employs H_2_ as the reducing agent, effectively avoiding contamination from transition metal chalcogenides and simultaneously enhancing the growth yield.^[^ ^18^, ^20^ ^]^ This chalcogen‐free APCVD strategy facilitates scalable, residue‐free synthesis of high‐quality (100)‐oriented MoO_2_ nanoribbon arrays.

Figure S2a (Supporting Information) shows that 2 cm × 2 cm large‐area metallic (100)‐oriented MoO_2_ nanoribbon arrays have been successfully synthesized on *c*‐sapphire substrate via the APCVD method. Figure 1c shows that the as‐grown MoO_2_ nanoribbons are densely and uniformly distributed across the entire substrate and vertically aligned on the *c*‐sapphire surface (inset). After transfer onto a SiO_2_/Si substrate, their well‐defined ribbon morphology is retained (Figure S2b,c, Supporting Information). Statistical analysis reveals that the nanoribbons exhibit typical lengths of ≈50 µm and widths of ≈2.5 µm. Atomic force microscopy measurements confirm their smooth surface morphology with a root‐mean‐square roughness below 0.5 nm and thicknesses ranging from 20 to 50 nm (Figure S3, Supporting Information). To investigate the crystal structure and chemical composition of the as‐grown nanoribbons, Raman spectroscopy and transmission electron microscopy (TEM) were employed. As shown in Figure S4a (Supporting Information), distinct Raman peaks characteristic of monoclinic MoO_2_ are observed, corresponding to the A_g_‐δ(OMo_2_), A_g_‐δ(OMo_2_), A_g_‐δ(OMo_3_), A_g_‐δ(O═Mo_3_), B_1g_‐ν(OMo_3_), and B_3g_‐δ vibrational modes.^[^ ^21^, ^22^ ^]^ The low‐magnification TEM image in Figure 1d reveals that the nanoribbon exhibits high transparency, indicating its ultrathin thickness. The corresponding fast Fourier transform (FFT) pattern (inset of Figure 1d) displays sharp diffraction spots, confirming the single‐crystalline nature of the nanoribbon. As shown in Figure 1e, the measured interplanar spacings are ≈0.477 and 0.276 nm along the [020] and [10$\bar{2}$] directions, respectively, with an interaxial angle *α* close to 90°. These values are consistent with the lattice parameters of the (100) plane of monoclinic MoO_2_ with space group *P21/c*, as corroborated by density functional theory (DFT) calculations (Figure 1f).

Further structural confirmation is provided by the X‐ray diffraction (XRD) pattern (Figure S4b, Supporting Information), where all diffraction peaks match well with the reference pattern of monoclinic MoO_2_ (JCPDS No. 97‐002‐3722). High‐angle annular dark‐field scanning transmission electron microscopy (HAADF‐STEM) and energy‐dispersive X‐ray spectroscopy (EDX) elemental mapping (Figure 1g–i) reveal a uniform distribution of Mo and O throughout the nanoribbon, with a Mo:O atomic ratio of ≈1:2. Collectively, the Raman, TEM, XRD, and EDX results unambiguously confirm that the nanoribbons are single‐crystalline, (100)‐oriented monoclinic MoO_2_.

The electronic band structure of the (100)‐oriented MoO_2_ nanoribbon, calculated using the Perdew–Burke–Ernzerhof (PBE) functional, is presented in Figure S5 (Supporting Information), revealing its intrinsic gapless metallic nature under ambient stress‐free conditions. Based on this, the average electron mobility of the as‐grown (100)‐oriented MoO_2_ nanoribbons is determined to be 664.13 cm^2^ (V·s)^−1^ (Figure S6, Supporting Information), which is ≈2–4 times higher than those of black phosphorus (303 cm^2^ (V·s)^−1^) and MoS_2_ (183 cm^2^ (V·s)^−1^).^[^ ^23^ ^]^ Furthermore, electrical transport measurements of individual nanoribbons were conducted using a custom‐built microprobe system (Figure S7, Supporting Information). The average electrical conductivity (*σ*) of a single (100)‐oriented MoO_2_ nanoribbon is estimated to be ≈10^7^ S m^−1^, significantly outperforming previously reported (100)‐oriented MoO_2_ (≈10^5^ S m^−1^),^[^ ^18^ ^]^ as well as many typical nanomaterials, including black phosphorus (10^3^–10^4^ S m^−1^),^[^ ^24^ ^]^ MoS_2_ (10^−5^–10^−2^ S m^−1^),^[^ ^25^ ^]^ and WS_2_ (≈10^−4^ S m^−1^).^[^ ^26^ ^]^ Notably, its conductivity is comparable to that of high‐quality graphene (≈10^6^ S m^−1^).^[^ ^27^ ^]^

The light absorption properties of the (100)‐oriented MoO_2_ nanoribbons were systematically investigated using Fourier Transform Infrared (FTIR) spectroscopy. As shown in Figure 1j, the nanoribbons exhibit an average absorbance exceeding 20% across an ultrabroad spectral range of 3–16 µm, significantly outperforming their (010)‐oriented counterparts (as depicted in Figure S8, Supporting Information) and display absorbance below 1% over the same range. This pronounced facet‐dependent optical response is further supported by first‐principles calculations (Figure S9a, Supporting Information), where the theoretical absorbance of the (100)‐oriented MoO_2_ consistently exceeds 20% throughout the mid‐ to long‐wavelength infrared regime. Correspondingly, the transmittance of the (100)‐oriented nanoribbons exceeds 65%, while the reflectance remains below 2% across the same range (Figure S9b, Supporting Information). These results underscore the advantages of the (100) crystallographic orientation in promoting strong light–matter interaction, high absorption, and enhanced photocurrent generation. In conjunction with their excellent metallic conductivity and high carrier mobility, (100)‐oriented MoO_2_ nanoribbons synthesized via APCVD present a compelling platform for next‐generation, broadband, self‐powered optoelectronic devices.

### 2.2 Photoresponse and Mechanism in (100)‐Oriented MoO_2_ Nanoribbon Photodetectors

**Figure 2:**
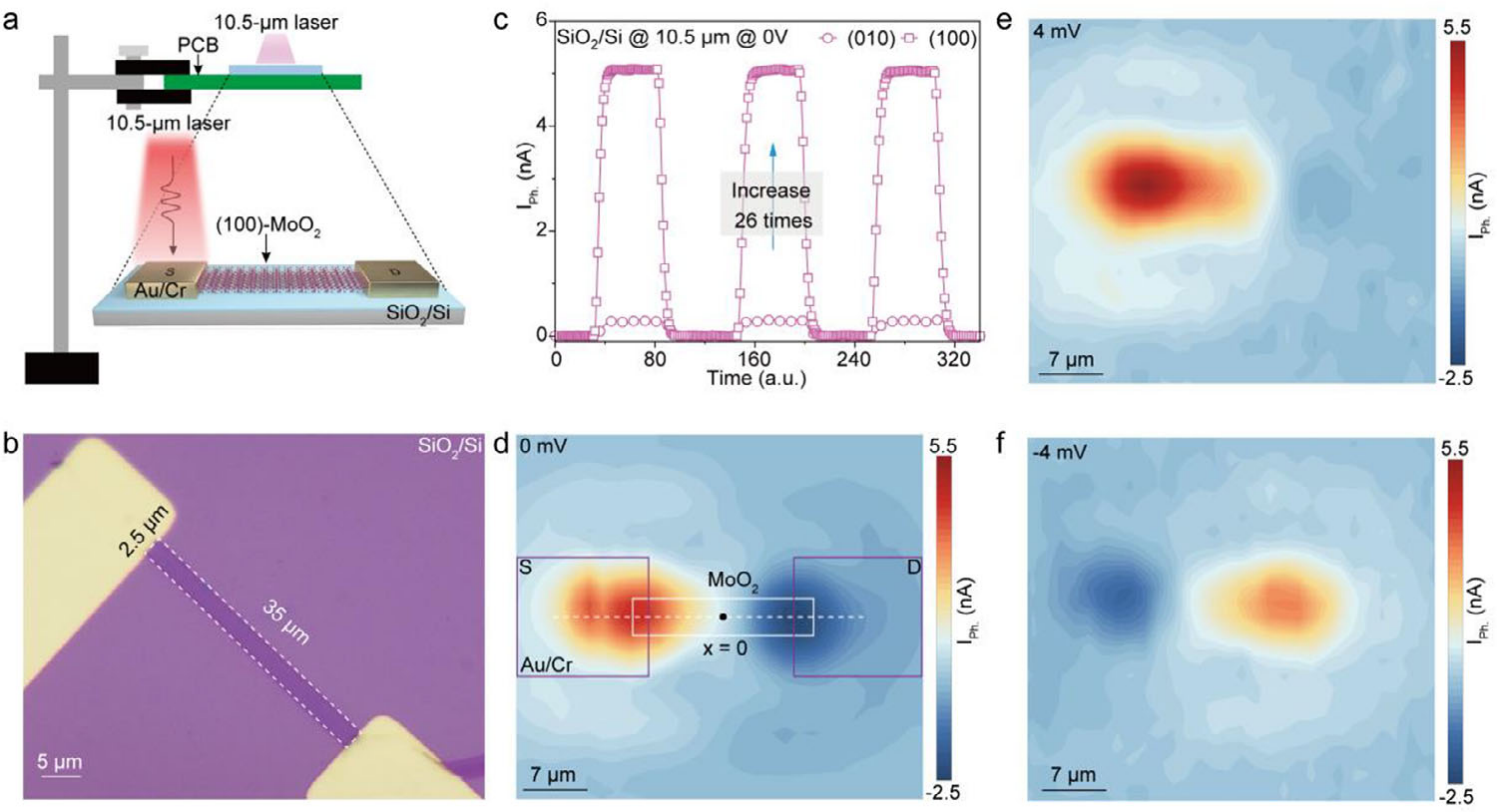
Photoresponse behaviors and physical mechanism of individual (100)‐oriented MoO_2_ photodetector on SiO_2_/Si substrate. a,b) Schematic diagram (a) and optical microscope image (b) individual (100)‐oriented MoO_2_ nanodevice, respectively, where *S* and *D* represent the source and drain electrodes. c) Zero‐biased photocurrent (*I_Ph._*)‐time curves. d–f) Photocurrent spatial distribution images of the photodetector at 0 (d), +4 (e), and −4 mV (f), respectively. For collecting the photocurrent time‐resolved responses and spatial images, the irradiation wavelength is 10.5 µm, with a light power density of 2.40 × 10^4^ mW cm^−2^.

To investigate the optoelectronic characteristics of the synthesized nanoribbons, photodetector devices based on individual (100)‐oriented MoO_2_ nanoribbons were fabricated. **Figure** **2**a shows the schematic diagram of the measurement system (For more details, please refer to the photodetection measurements in the Experimental Section) and the device structure of an individual MoO_2_ photodetector, while Figure 2b shows the corresponding optical microscopy image. In the device configuration, 120‐nm Au/10‐nm Cr films were deposited as source and drain electrodes with an interelectrode spacing of ≈35 µm. The selected MoO_2_ nanoribbon has a width of ≈2.5 µm and a thickness of ≈30 nm. The photocurrent response of the (100)‐oriented MoO_2_ nanoribbons under zero bias is ≈26 times higher than that of the (010)‐oriented MoO_2_ nanoribbons under identical 10.5 µm illumination (Figure 2c), confirming the decisive role of the (100) facet in enabling efficient broadband photoelectric conversion. To spatially resolve the photocurrent generation and probe the underlying photoresponse mechanism, scanning photocurrent microscopy was conducted, as shown in Figure 2d. Here, the metal electrodes and the nanoribbon channel are marked by pink and white boxes, respectively. A mid‐infrared laser with a wavelength of 10.5 µm and a spot diameter of ≈43.87 µm was used for illumination, with the incident power density varied from 5.95 × 10^3^ to 2.4 × 10^4^ mW cm^−^^2^. During the measurements, a small bias voltage (*V*_SD_) ranging from −4 to +4 mV was applied, and the detector was scanned below the laser spot with a step of 2 µm.

The photocurrent image of the (100)‐oriented MoO_2_ nanodevice at *V*_SD_ = 0 mV is shown in Figure 2c, where a self‐powered photocurrent with an uneven spatial distribution along the nanoribbon axis is clearly observed. The asymmetric photocurrent distribution in zero‐biased photodetectors is commonly attributed to either the photovoltaic effect (PVE) or the photothermoelectric effect (PTE). Given that the (100)‐oriented MoO_2_ nanoribbon exhibits metallic behavior, Ohmic contacts are expected to form at the interface between the Cr electrodes and the nanoribbon, which excludes the PVE as the dominant mechanism responsible for the self‐powered photocurrent in our devices. Instead, the PTE provides a more plausible explanation. In this mechanism, the self‐powered photocurrent arises from a photogenerated voltage induced by a temperature gradient between the Cr electrode and the (100)‐oriented MoO_2_ nanoribbon, caused by their differing Seebeck coefficients. The PTE photocurrent (*I*_Ph_) can be described by the equation:^[^ ^28^ ^]^

(1) $$I_{Ph.}=\frac{\left(S_{2}\;-\;S_{1}\right)\Delta T}{R}$$

where *S*_1_ and *S*_2_ are the Seebeck coefficients of the MoO_2_ nanoribbon and the Cr electrode, respectively; Δ*T* is the temperature difference between the illuminated region and its surroundings; and *R* is the electrical resistance of the nanoribbon. The temperature gradient Δ*T* can be further expressed as:

(2) $$\Delta T=\frac{P\alpha }{\pi kd}$$

where *P* is the incident light power, *α* is the light absorbance, *k* is the thermal conductivity, and *d* is the thickness of the nanoribbon. Combining these expressions, the PTE photocurrent is given by:

(3) $$I_{Ph.}=\frac{\left(S_{2}\;-\;S_{1}\right)P\alpha }{\pi kdR}$$

indicating that the PTE photocurrent increases with the incident light power, absorbance, and the difference in Seebeck coefficients between the nanoribbon and electrode, but decreases with increasing thermal conductivity and electrical resistance of the nanoribbon. Consistent with this model, evident photocurrents of 4.52 and 2.39 nA are observed near the two Cr/MoO_2_ interfaces at the source and drain electrodes under illumination (Figure 2c; Figure S10, Supporting Information). In particular, within the channel region, the photocurrent undergoes a transition from positive to negative as the laser spot is scanned from the source electrode to the drain electrode. The photocurrent disappears entirely when the laser spot is positioned at the midpoint of the channel (*x* = 0, Figure 2c; Figure S10, Supporting Information). Collectively, these observations strongly support that the zero‐bias photoresponse of the (100)‐oriented MoO_2_ nanodevice is governed by the PTE.

As shown in Figure 2e and Figure S10 (Supporting Information), both the source and drain photocurrents of the (100)‐oriented MoO_2_ nanoribbon photodetector increase gradually with increasing *V*_SD_ from 0 to +4 mV. To elucidate the observed variations in photocurrent behavior, we propose the following mechanism. While the photobolometric effect (PBE) may contribute to the photocurrent in biased photodetectors fabricated on thermally insulating substrates, it cannot account for the photoresponse under zero‐bias conditions—where the PTE dominates. Therefore, the photocurrent observed in the biased (100)‐oriented MoO_2_ device likely arises from the interplay between the electric field‐driven drift current and the thermoelectric current generated by the PTE. The photosensitivity of the metallic photobolometric detector is usually determined the thermal resistance (*R_t_*) as:^[^ ^29^, ^30^ ^]^

(4) $$R_{t}=R_{0}\left[1+\alpha \left(t\;-\;t_{0}\right)\right]$$

where *t* denotes the measurement temperature, *R*_0_ is the electric resistance at the reference temperature *t*_0_, and *α* represents the temperature coefficient of electrical resistance. Specifically, when illuminated under a +4 mV bias, the photocurrent exhibits only minor changes compared to the zero‐bias condition (Figure S10, Supporting Information), indicating that the contribution from the PBE is minimal in this configuration. This is further supported by the measured resistance temperature coefficient of the metallic (100)‐oriented MoO_2_ nanoribbon, which is relatively low (0.124) (Figure S11, Supporting Information). Additionally, the high thermal conductivity (≈100 W mK)^−1^)^[^ ^31^ ^]^ of the underlying SiO_2_/Si substrate promotes rapid heat dissipation, minimizing local heating effects and resulting in only a slight increase in device resistance with applied bias. These findings collectively suggest that the influence of the PBE on the biased‐photocurrent is negligible under our experimental conditions.

Given that the direction of the electric field‐driven current is dictated by the polarity of the applied bias, the spatial distribution of the total photocurrent along the nanoribbon becomes increasingly asymmetric with bias, as evident in Figure 2e and Figure S10 (Supporting Information). Upon reversing the applied bias from +4 to −4 mV, the polarity of both the source and drain photocurrents is reversed, as clearly shown in Figure 2f and Figure S10 (Supporting Information). Specifically, at −4 mV bias, the drain photocurrent reaches a maximum of 3.28 nA, while the source photocurrent exhibits a minimum of −1.45 nA. This behavior can be understood as the result of opposing contributions from the drift current and the thermoelectric current generated at the source electrode, with the latter decreasing along the nanoribbon axis from source to drain. Consequently, the observed polarity reversal between ±4 mV confirms that the photocurrent in the biased (100)‐oriented MoO_2_ nanoribbon device on a SiO_2_/Si substrate is primarily governed by the PTE.

### 2.3 Ultrabroadband Self‐Powered Photoresponses of an Individual (100)‐Oriented MoO_2_ Photodetector on a SiO_2_/Si Substrate

**Figure 3:**
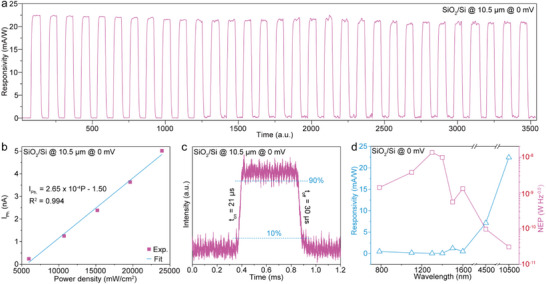
The ultrabroad band photoresponse performances of individual (100)‐oriented MoO_2_ photodetector on SiO_2_/Si substrate. a) Time‐resolved photoresponse of the photodetector during 31 repeated illumination cycles. b) Dependence of the photocurrent on light power density, with the power density ranging from 5.95 × 10^3^ to 2.40 × 10^4^ mW cm^−2^. c) The switching characteristic of the photodetector. d) Responsivity (blue) and *NEP* (red) of the photodetector as a function of illumination wavelength. For all of the measurements, the photodetector operates in a zero‐biased mode.

To investigate the broadband self‐powered photoresponse characteristics of the (100)‐oriented MoO_2_ nanoribbon on a SiO_2_/Si substrate, a series of lasers with wavelengths ranging from 0.78 to 10.5 µm were employed as irradiation sources. The incident light power was tunable from 1.04 to 863 µW to enable systematic evaluation. A key performance metric of photodetectors, the photoresponsivity (*R*_Ph._), was calculated using the relation as:^[^ ^32^ ^]^

(5) $$R_{Ph.}=\frac{I_{Ph.}\;-\;I_{Dark}}{PS}$$

where *I*_Ph._ and *I*_Dark_ are the photocurrent and dark current, respectively, while *P* and *S* denote the incident light power density and effective illumination area. **Figure** **3**a presents the long‐term photoresponsivity behavior of an individual zero‐biased (100)‐oriented MoO_2_ nanodevice under 10.5 µm illumination at an excitation power density of 2.40 × 10^4^ mW cm^−2^. Notably, the nanoribbon device achieves a high photoresponsivity of 22 mA W^−1^ and exhibits excellent operational stability, with a mean response fluctuation of less than 0.21% over 31 continuous measurement cycles. The photocurrent shows a clear linear dependence on the incident light power density, as shown in Figure 3b and Figure S12 (Supporting Information), following the relation *I*_Ph._∝ 2.65 × 10^−4^ *P*. Further temporal response analysis (Figure 3c) reveals fast rise (*t*_on_) and decay times (*t*_off_) of 21 and 30 µs, respectively. This rapid photoresponse is attributed to the PTE mechanism mediated by the high electron mobility of the metallic (100)‐oriented MoO_2_ nanoribbon. Beyond single‐wavelength operation, the device also exhibits robust broadband photoresponse. As illustrated in Figure S13 (Supporting Information), the zero‐biased MoO_2_ nanoribbon maintains stable and efficient photosensitivity from the visible (0.78 µm) to the far‐infrared (10.5 µm), under tunable excitation powers ranging from 1.04 to 863 µW.

*NEP* and photodetectivity (D^*^) are other key figures of merit for evaluating the sensitivity of photodetectors, as it quantify the minimum detectable optical power under unit bandwidth. They are defined as:^[^ ^11^, ^33^, ^34^ ^]^

(6) $$NEP=\frac{I_{n}}{R_{Ph.}}$$

(7) $$D^{*}=\frac{\sqrt{AB}}{NEP}$$

where *I_n_*, *R_Ph._* and *A* respectively denote thermal current noise of the ohmic‐contact type photodetector (more calculation details can be seen in the photodetection measurements of the Experimental Section), the photoresponsivity and the effective area of the device, and *B* is the bandwidth. The photoresponsivity *R_Ph._* of the nanoribbon device is seen to exhibit a gradual increase tendency with the irradiation wavelength (Figure 3d), which can be ascribed to its irradiation wavelength‐dependent absorbance (Figure S9b, Supporting Information). Also, the noise power spectra of the zero‐biased (100)‐oriented MoO_2_ photodetector were then obtained under various illumination wavelengths (Figure 3d; Figure S14, Supporting Information). Nonetheless, the detector consistently achieves responsivity values exceeding 0.07 mA W^−1^ across a broad spectral range from the visible (0.78 µm) to the far‐infrared (10.5 µm). Correspondingly, the *NEP* values remain below 3.75 × 10^−8^ W Hz^−0.5^ throughout this range, indicating high sensitivity under self‐powered operation. Notably, under 10.5 µm excitation, the (100)‐oriented MoO_2_ nanodevice achieves a record‐low *NEP* of 3.08 × 10^−11^ W Hz^−0.5^, accompanied by a peak photoresponsivity of 22.4 mA W^−1^—the lowest *NEP* reported to date among all self‐powered photodetectors operating at this wavelength (Table S1, Supporting Information). This outstanding sensitivity underscores the superior performance of metallic MoO_2_ nanoribbons in long‐wavelength infrared detection. A comparative analysis of recently reported zero‐biased nanoscale photodetectors (Table S1, Supporting Information) further highlights the exceptional figures of merit achieved by our device. Although the D^*^ value of the (100)‐MoO_2_ photodetector is not as high as several nanomaterial‐based photodetectors with remarkable performances, it can simultaneously have a very high photoresponsivity of 22.4 mA W^−1^ in a wide wavelength range from 0.78 to 10.5 µm, a short switching time of ≈20 µs and a much low *NEP* value of 3.8 × 10^−11^ WH_Z_^−0.5^, surpassing many other nanoscale self‐powered photodetectors. Most of all, the (100)‐oriented MoO_2_ photodetectors have successfully solved the contractionary between rapid switching speed and high photoresponsivity for most self‐powered broadband photodetectors. By comprehensively assessing the photoresponse performances of the (100)‐oriented MoO_2_ photodetector, it should have great potential in advanced self‐powered nanoscale photodetectors.

### 2.4 Photoresponse Characteristics of Individual (100)‐Oriented MoO_2_ Photodetector on Flexible PET Substrates

**Figure 4:**
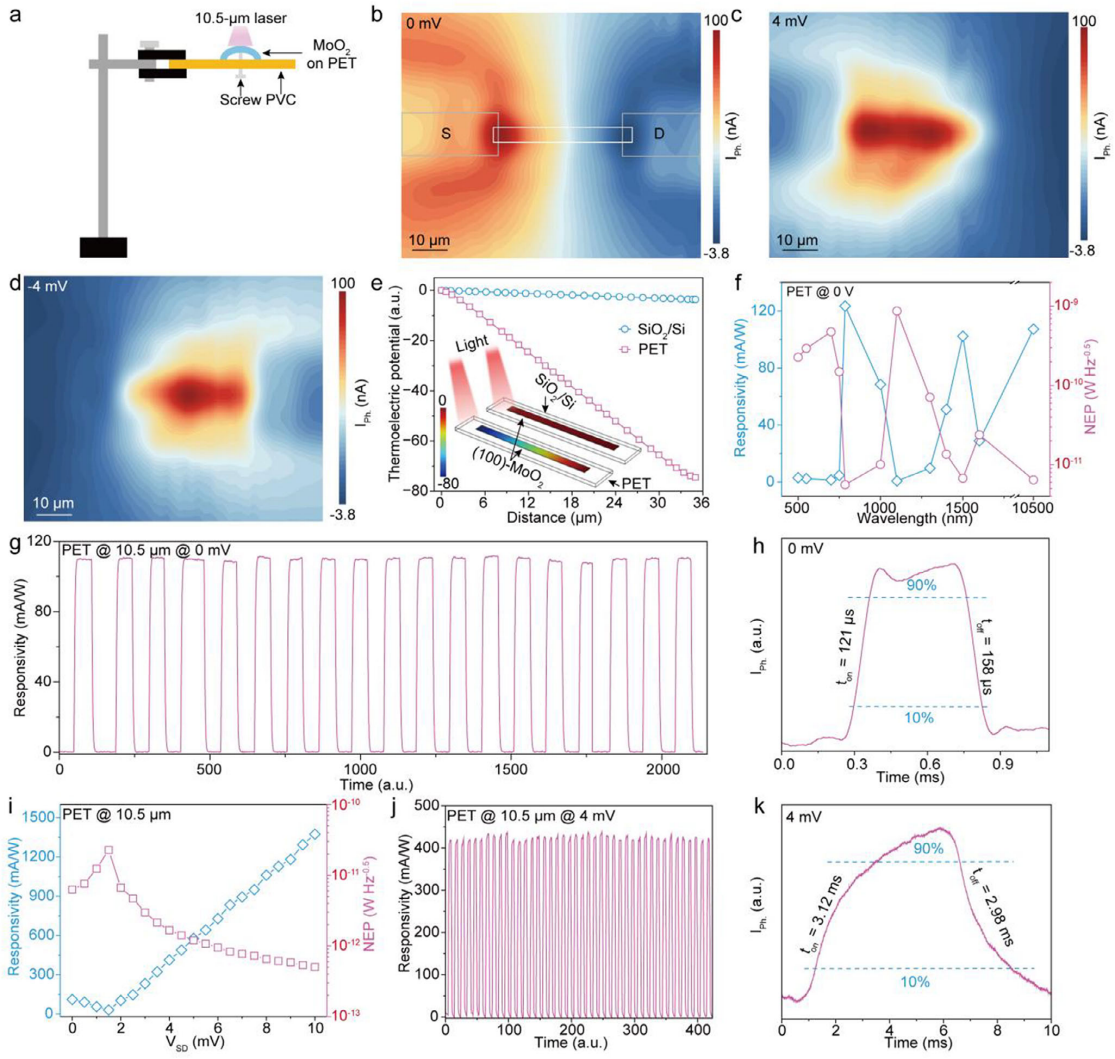
Photoresponses of individual (100)‐oriented MoO_2_ photodetector on PET substrate. a) Schematic diagram of the photodetection system of individual (100)‐oriented MoO_2_ nanodevice on PET substrate. b–d) Scanning photocurrent distribution images of the (100)‐oriented MoO_2_ nanoribbon on PET substrate under 10.5‐µm irradiation, in which the bias voltage is 0 (b), 4 (c), and −4 mV (d), respectively. e) Comparison of the simulating thermoelectric potential distribution of individual (100)‐oriented MoO_2_ nanoribbons on SiO_2_/Si substrate with that on PET substrate. f) The photoresponsivity and *NEP* values of the zero‐biased photodetector on PET substrate under different excitation wavelengths. g) Time‐resolved photoresponse of the photodetector during 19 repeated illumination cycles. h,k) The switching characteristics of the photodetector under 0 mV‐ (h) and 4 mV‐ (k) bias, respectively. i) Photoresponsivity and *NEP* values of the photodetector at different biases. j) Time‐resolved photoresponse of the 4 mV‐biased photodetector during 42 repeated illumination cycles.

To evaluate the practical applicability of (100)‐oriented MoO_2_ nanoribbons in flexible and wearable optoelectronic devices, we fabricated individual photodetectors on flexible PET substrates, as illustrated in Figure S15 (Supporting Information). For device fabrication, 120‐nm Au/10‐nm Cr electrodes were thermally evaporated onto both ends of the nanoribbon, defining an electrode spacing of 50 µm. The nanoribbon itself exhibits a width of ≈2 µm and a thickness of ≈35 nm. During measurement, a bias voltage ranging from 0 to 10 mV was applied, and a 10.5‐µm laser with a tunable power density from 5.95 × 10^3^ to 2.4 × 10^4^ mW cm^−2^ served as the excitation source. **Figure** **4**a schematically illustrates the photodetection system individual (100)‐oriented MoO_2_ nanodevice on a PET substrate (For more details, please refer to the photodetection measurements of the Experimental Section). To investigate the spatial photoresponse behavior and underlying mechanism, photocurrent distribution maps of the (100)‐oriented MoO_2_ photodetector on PET were acquired at three different bias conditions: 0, +4, and –4 mV (Figure 4b–d). Under zero bias (Figure 4b), the photocurrent distribution exhibits a symmetric profile with polarity reversal across the nanoribbon, consistent with the response observed in devices fabricated on SiO_2_/Si substrates (Figure 2c). This characteristic spatial distribution strongly indicates that the PTE remains the dominant mechanism even on the flexible PET substrate. These findings collectively demonstrate that the (100)‐oriented MoO_2_ nanoribbon retains its intrinsic PTE‐governed photoresponse when transferred to flexible substrates, underscoring its strong potential for integration into next‐generation wearable and flexible broadband photodetectors.

Upon increasing the applied voltage to +4 mV, the photocurrent of the (100)‐oriented MoO_2_ photodetector on the PET substrate becomes uniformly distributed along the nanoribbon axis, reaching a peak value of 99 nA (Figure 4c; Figure S16, Supporting Information). Notably, this photocurrent is substantially higher than that observed at the nanoribbon–electrode contact region (18.1 nA), indicating a spatially homogeneous carrier generation and transport along the channel. When the applied voltage is reversed to –4 mV (Figure 4d), the photocurrent distribution remains nearly identical to that under positive bias, a behavior markedly distinct from the polarity‐sensitive distribution observed in devices on SiO_2_/Si substrates (Figure 2e,f). This contrast strongly suggests that the underlying photoresponse mechanisms differ between the two substrate platforms, especially for biased operation mode. As previously discussed, the high thermal conductivity of the SiO_2_/Si substrate (≈100 W (mK)^−1^)^[^ ^31^ ^]^ enables efficient dissipation of Joule heat, thereby suppressing the PBE and favoring the PTE as the dominant mechanism in biased MoO_2_ devices. In contrast, the PET substrate possesses a significantly lower thermal conductivity (≈0.04 W (mK)^−1^),^[^ ^35^ ^]^ which limits heat dissipation and allows local temperature to rise substantially under bias due to Joule heating (Figure S17, Supporting Information). This thermal accumulation enhances the PBE, which increasingly dominates the photocurrent response as the bias voltage increases.

In PBE‐governed operation, the photogenerated current is driven primarily by temperature‐induced changes in carrier mobility and resistivity, rather than by thermoelectric gradients. Since Joule heating dominates over photothermal heating from the incident light, the resulting photocurrent tends to distribute uniformly across the nanoribbon, independent of the bias polarity. This behavior aligns with the experimental photocurrent distribution maps obtained on the PET substrate (Figure 4c,d), which show a markedly different profile compared to the PTE‐dominated response on the SiO_2_/Si substrate—characterized by intensity‐symmetric yet polarity‐reversed signals (Figure 2e,f). It is clearly seen that the slope of the *I–V* curve under irradiation occurs to have a decrease than it under 10.5‐µm dark conditions, in which the electrical resistance of the nanoribbon device increases from 174.16 to 178.59 Ω (Figure S18, Supporting Information). It further proves that the photoresponse mechanism of the biased MoO_2_ nanodevice on the PET substrate should be attributed to the PBE. Therefore, it can be concluded that under bias, the photoresponse mechanism of the (100)‐oriented MoO_2_ nanodevice transitions from PTE on rigid SiO_2_/Si substrates to PBE on flexible PET substrates. The enhanced photoresponse of the photodetector on the PET substrate under zero bias—relative to that on the SiO_2_/Si substrate—can be attributed to local heat accumulation due to the low thermal conductivity of PET. Numerical simulations of the thermoelectric potential distribution under localized illumination (Figure 4e, inset) reveal that the poor thermal conductivity of PET leads to significant heat retention at the illumination site. This results in a steeper thermoelectric potential gradient between the illuminated region and the device termini, thereby generating a substantially stronger PTE‐induced current. In contrast, the higher thermal conductivity of the SiO_2_/Si substrate facilitates efficient heat dissipation, limiting thermal accumulation and reducing the resulting potential gradient (Figure 4e). This comparison highlights the crucial role of substrate thermal properties in modulating the self‐powered photoresponse behavior of the device.

Furthermore, the broadband photoresponse characteristics of the zero‐biased (100)‐oriented MoO_2_ nanodevice on a flexible PET substrate are evaluated across a wide spectral range from 500 nm to 10.5 µm, as shown in Figure 4f and Figures S19,S20 (Supporting Information). Under 10.5‐µm irradiation, the device exhibits its highest *NEP* of 6.64 pW Hz^−0.5^ and D^*^ of 4 × 10^7^ Jones, along with the second‐highest *R*_Ph._ of 107.32 mA W^−1^ among all tested wavelengths. These results suggest that the nanoribbon device achieves its optimal self‐powered photodetection performance in the LWIR regime, making it particularly promising for broadband and mid‐to‐far infrared nanoscale photosensitive applications. The stability of this photoresponse is further demonstrated in Figure 4g, where the device exhibits highly reproducible switching behavior with minimal fluctuation (<0.02%) over 19 consecutive on–off cycles under 10.5‐µm illumination. Temporal response measurements (Figure 4h) indicate a rise time of 121 µs and a fall time of 158 µs, confirming fast and reliable operation in the zero‐bias condition. The self‐powered photoresponse performances of the (100)‐MoO_2_ photodetector on a PET substrate are compared with those of other nanoscale photodetectors on a flexible substrate in Table S2 (Supporting Information). It is clearly seen that the (100)‐MoO_2_ photodetector on the PET substrate nearly has similar excellent photoresponse performances with it on the SiO_2_/Si substrate in a very broadband wavelength range. Most of all, the (100)‐oriented MoO_2_ photodetectors have successfully solved the contractionary between rapid switching speed and high photoresponsivity for most broadband self‐powered photodetectors. By comprehensively assessing the photoresponse performances of the (100)‐oriented MoO_2_ photodetector, it should have great potential in advanced self‐powered nanoscale photodetectors.

Power density‐dependent measurements under 10.5‐µm excitation (Figure S21, Supporting Information) reveal that the PET‐supported nanodevice possesses a lower minimum detectable power density (4.37 × 10^3^ mW cm^−2^) compared to its SiO_2_/Si‐supported counterpart (5.65 × 10^3^ mW cm^−2^), further confirming its superior sensitivity. Compared to the zero‐biased MoO_2_ device on a SiO_2_/Si substrate (*NEP* = 30.8 pW Hz^−0.5^, *R*_Ph._ = 22 mA W^−1^, rise/fall time = 21/30 µs), the PET‐supported photodetector shows substantial improvement in photoresponsivity and noise performance. The only trade‐off is a modest increase in response time, which can be rationalized by the distinct photoresponse mechanisms governed by the substrate thermal properties. Specifically, the PBE‐dominated behavior in the PET‐supported device leads to higher local temperatures (Figure S17, Supporting Information), which enhances carrier–phonon scattering and slightly prolongs the switching dynamics. Nonetheless, the overall enhancement in responsivity and detection limit underscores the ability of the PET substrate to boost the photothermal conversion efficiency and promote superior self‐powered photodetection across a broad spectral range.

Under 10.5 µm irradiation, the *R*_Ph._ of the individual (100)‐oriented MoO_2_ detector on the PET substrate exhibits a nonmonotonic dependence on the *V*_DS_, as shown in Figure 4i and Figure S22 (Supporting Information). Specifically, *R*_Ph._ initially decreases from 107.31 to 27.63 mA W^−1^ as *V*_DS_ increases from 0 to +4 mV, but subsequently rises sharply to a peak value of 1370 mA W^−1^ at +10 mV. This nonmonotonic behavior is likely governed by the interplay between two competing effects: the suppression of carrier mobility at elevated local temperatures due to increased carrier–phonon scattering, and the enhancement of carrier drift velocity under stronger electric fields.^[^ ^10^, ^36^, ^37^, ^38^, ^39^ ^]^ At the maximum photoresponsivity of 1370 mA W^−1^, the device also achieves an ultralow *NEP* of 0.95 pW Hz^−0.5^ and a very high D^*^ of 2.8 × 10^8^ Jones, indicating exceptional detectivity in the low‐bias regime. The photoresponse stability under low bias is further verified in Figure 4j, where the +4 mV‐biased device exhibits highly repeatable behavior with average fluctuations below 0.01% over 42 continuous measurement cycles under 10.5 µm illumination.

Temporal response characterization (Figure 4k) reveals that the rise and fall times of the 4 mV‐biased device extend to 3.12 and 2.98 ms, respectively—approximately 20 times longer than those of the zero‐biased device (121 and 158 µs, Figure 4h). This pronounced slowdown in response speed is attributed to the predominance of the PBE under applied bias (Figure S17, Supporting Information). Unlike the prompt carrier diffusion in PTE‐driven processes, the PBE mechanism necessitates a finite time for local heat accumulation to modulate conductivity. Additionally, the elevated device temperature under bias enhances phonon scattering, which further impedes the transport of photogenerated carriers and prolongs the device's switching dynamics. The corresponding light power density‐dependent photocurrent response is shown in Figure S23 (Supporting Information), where the minimum detectable power density for the biased device is reduced to 2.33 × 10^3^ mW cm^−2^—approximately half that of the self‐powered device (4.37 × 10^3^ mW cm^−2^). These comparative results confirm that the application of a modest external bias can substantially enhance most photoresponse metrics, including responsivity and sensitivity. However, this improvement comes at the expense of slower response speed, primarily due to the thermally induced mobility degradation.^[^ ^37^ ^]^ Overall, the findings highlight a tunable trade‐off between performance and response time, offering flexible optimization strategies for MoO_2_‐based infrared photodetectors integrated on low‐thermal‐conductivity substrates.

And the underlying mechanism of the outstanding photoresponse performances of the (100)‐oriented MoO_2_ photodetector is proposed as follows. One is that the (100)‐oriented MoO_2_ nanoribbon has metallic electrical conductivity of ≈10^7^ S m^−1^, very large carrier mobility of 664.14 cm^2^ V^−1^ s^−1^ and nice ambient stability in comparison with many other nanomaterials (e.g., 80 cm^2^ V^−1^ s^−1^@10^−5^–10^−2^ S m^−1^ for MoS_2_,^[^ ^25^, ^40^ ^]^ ≈48 cm^2^ V^−1^ s^−1^@≈10^−4^ S m^−1^ for WS_2_,^[^ ^26^, ^41^ ^]^ etc.), which are very favorable to enhance the photoresponse wavelength range, switching speed and photodetection sensitivity of the photodetectors. The second is that the (100)‐oriented MoO_2_ nanoribbon has a much higher light absorbance (>20%) than most of the excellent photosensitive nanomaterials in a broad spectral range from visible to far‐infrared band (e.g., <4% for MoS_2_ and WS_2_ in the near‐infrared range,^[^ ^42^ ^]^ ≈2.3% for graphene in near‐infrared range,^[^ ^43^ ^]^ etc.). The last is that there is a strong light–matter interaction existing on the (100) surface of MoO_2_ due to the high asymmetric characteristic of the atomic arrangement, which is beneficial to improve their photodetection performances. Owing to the above merits of the (100)‐oriented MoO_2_ nanoribbon, it exhibits more exceptional ultrabroadband photoresponse behaviors than many other excellent nanomaterial‐based photodetectors.

To evaluate the ultrabroadband photodetection capabilities of the flexible, self‐powered (100)‐oriented MoO_2_ detector under mechanical deformation, its photoresponse characteristics were examined at varying bending angles (Figure S24, Supporting Information). Remarkably, across a wide range of incident wavelengths, the zero‐biased photodetector maintains stable and high‐performance photoresponse even under significant bending. In particular, under all three representative excitations, the device exhibits nearly identical responsivity and signal stability compared to the unbent state, even at bending angles as large as 60°. These results affirm the mechanical robustness and operational reliability of the flexible MoO_2_ photodetector. Taken together, the device demonstrates outstanding broadband self‐powered photodetection from 0.5 to 10.5 µm, underscoring its potential for integration into next‐generation wearable and deformable optoelectronic systems.

### 2.5 Long‐Wave Infrared Imaging Applications of the Self‐Powered (100)‐Oriented MoO_2_ Flexible Photodetector

**Figure 5:**
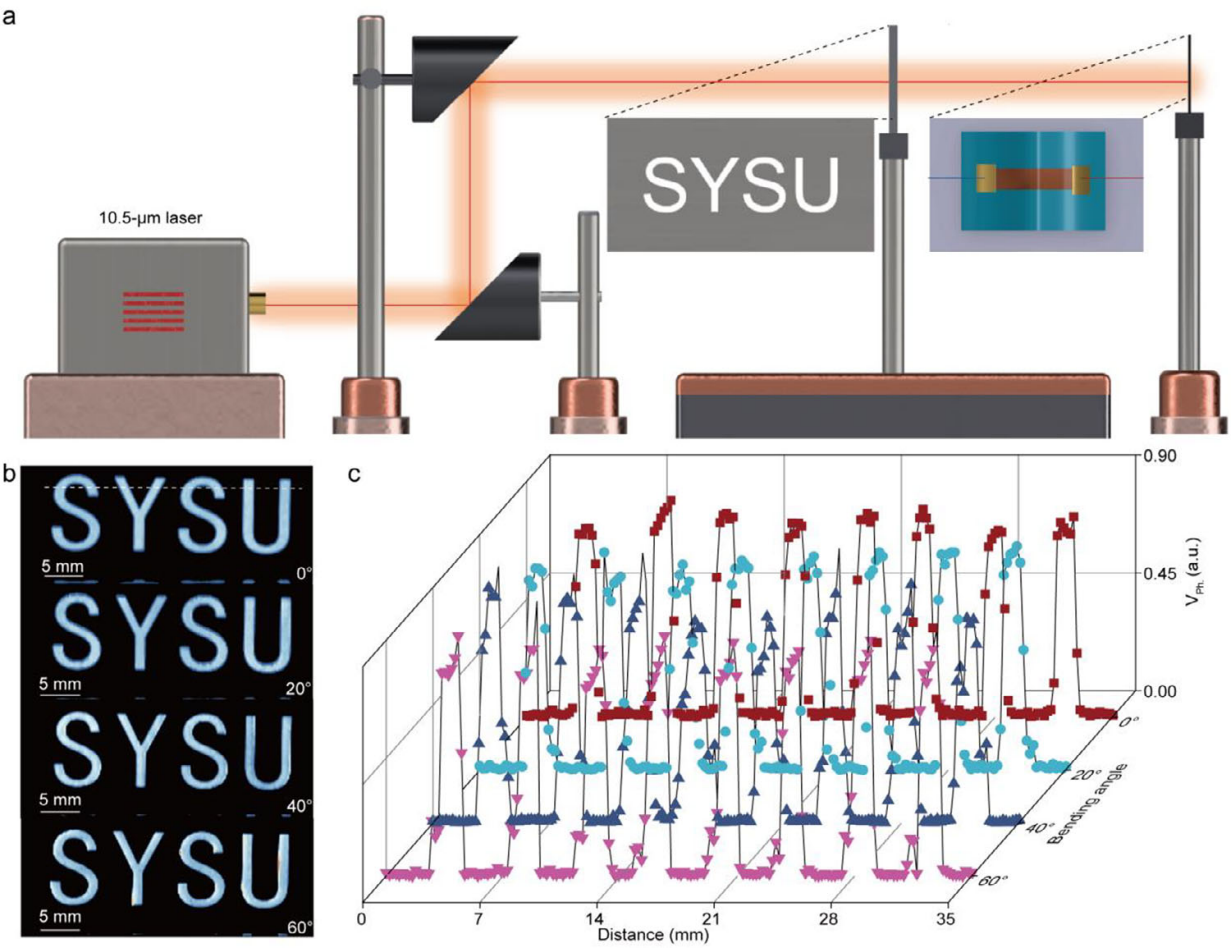
Long‐wave infrared imaging of the self‐powered flexible (100)‐oriented MoO_2_ photodetector. a) Schematic diagram of the LWIR scanning imaging system. b) The scanning images of the zero‐biased flexible photodetector with the bending angle from 0 to 60°. c) The corresponding photovoltage line profiles at different bending angles, extracted along the white dashed line in (b).

To further validate the practical applicability of the flexible (100)‐oriented MoO_2_ photodetector, its capability for long‐wave infrared (LWIR) imaging was evaluated under varying mechanical deformations. A far‐infrared imaging experiment was performed using an 81 × 191 pixel scan mode on the zero‐biased MoO_2_ photodetector integrated on a PET substrate. The imaging was conducted in situ at different bending angles to directly visualize the device performance under strain. As illustrated in **Figure** **5**a, a precision horizontal displacement stage equipped with a motor accurately positioning the metal mask bearing the target pattern. Incident LWIR radiation was transmitted through the patterned region to facilitate real‐time imaging by the self‐powered photodetector. During the measurements, a 10.5‐µm quantum cascade laser with an incident power density of 2.4 × 10^4^ mW cm^−2^ served as the irradiation source. Each pixel was recorded with an integration time of 0.5 s and a spatial scanning step of 0.3 mm. Figure 5b presents the reconstructed LWIR images acquired from the self‐powered MoO_2_ flexible photodetector at bending angles of 0°, 20°, 40°, and 60°. Notably, even at a maximum bending angle of 60°, the device consistently captured high‐fidelity images of the “SYSU” pattern, with well‐defined features and sharp contrast, underscoring its mechanical robustness and imaging integrity.

Furthermore, the distance‐dependent photovoltage distribution profiles shown in Figure 5c reveal near‐complete overlap across the bending angles, confirming that the device retains its exceptional photoresponse characteristics even under substantial mechanical deformation. This mechanical invariance can be attributed to the combination of the intrinsic flexibility of the PET substrate and the robust interfacial and electrical properties of the (100)‐oriented MoO_2_ nanoribbon, reinforcing its potential for integration into next‐generation wearable, uncooled LWIR imaging platforms.

## 3 Conclusion

In summary, we have successfully synthesized large‐area (100)‐oriented MoO_2_ nanoribbon arrays via a developed APCVD approach, demonstrating exceptional optoelectronic characteristics. Spectroscopic analysis and theoretical calculations reveal the outstanding optical properties of the nanoribbon, maintaining >20% absorbance and <2% reflectance across an ultrabroad 0.5–16 µm spectral range. Crucially, we identify a substrate‐dependent photoresponse transition: devices on high‐thermal‐conductivity SiO_2_/Si exhibit PTE dominance, while those on low‐thermal‐conductivity PET display PBE behavior. The flexible MoO_2_/PET photodetector achieves remarkable performance metrics, including a photoresponsivity of 107.31 mA W^−1^ and record‐low *NEP* of 6.64 pW Hz^−0.5^ at 10.5 µm illumination−surpassing all reported self‐powered photodetectors. This exceptional performance stems from the unique combination of metallic conductivity, high carrier mobility, and broadband absorption of the facet‐engineered (100)‐oriented MoO_2_ nanoribbon. Notably, the device maintains stable photoresponse under 60° mechanical bending, demonstrating robust flexibility across the visible to far‐infrared spectrum. These findings establish (100)‐oriented MoO_2_ nanoribbons as a promising candidate for next‐generation wearable broadband photodetection systems, while the facet engineering strategy provides a general framework for developing high‐performance metallic oxide optoelectronics.

## 4 Experimental Section

### Synthesis of (100)‐Oriented MoO_2_ Nanoribbons

The (100)‐oriented MoO_2_ nanoribbons were synthesized in a single‐zone tube furnace by the H‐APCVD way, and the detailed growth process was depicted as follows. First, the source materials were heated to 150 °C at a rising rate of 15 °C min^−1^ under Ar gas with a flow rate of 200 standard cubic centimeter per minute (sccm), and held here for 10 min. Second, the reaction boat was raised to 760 °C and kept here for 20 min, in which the mixed gas of 0.5 sccm H_2_ and 60 sccm Ar was introduced into the chamber. Finally, the furnace was cooled down to 600 °C with a rate of 20 °C min^−1^, and rapidly cooled down to room temperature with a dropping rate of ≈40 °C min^−1^ under the protection of 200 sccm Ar gas. For the (010)‐oriented MoO_2_ nanostructures, they could easily achieved by replacing the *c*‐sapphire substrate with SiO_2_/Si substate while keeping other growth parameters unvaried.^[^ ^44^ ^]^ By choosing different substrates, (010)‐oriented and (100)‐oriented MoO_2_ could be controllably prepared via the growth technique.

### Material Characterization

The surface morphology, chemical compositions, and crystalline structure of the (100)‐oriented MoO_2_ nanoribbons were examined using an optical microscope (OM, Olympus, BX53), XRD (D‐MAX 2200 VPC), X‐ray photoelectron spectroscopy (XPS, Thermofisher Nexsa), Raman spectroscopy (inVia Reflex, 532 nm laser), and transmission electron microscopy (TEM, FEI Titan 80‐300), respectively. And the thickness of the (100)‐oriented MoO_2_ nanoribbons was studied by atomic force microscope (Bruker Dimension Fastscan). The infrared absorbance spectrum of the sample was researched using the FTIR technique (Vertex 70).

### Fabrication of (100)‐Oriented MoO_2_ Nanoribbon Photodetector

The fabrication process of the photodetector was described in the following. First, the (100)‐oriented MoO_2_ nanoribbons were transferred from a sapphire substrate to a Si wafer coated with a 500‐nm‐thick SiO_2_ layer using a simple immersion and scraping method. 1) Some deionized (DI) water was dropped onto the nanoribbons on the sapphire substrate covered by the nanoribbon sample. 2) Tweezers were used to peel the nanoribbons out of the substate surface and immersed the nanoribbons into DI water. 3) The SiO_2_/Si substrate was immersed onto the DI water to adsorb (100)‐oriented MoO_2_ nanoribbons and annealed at 80 °C for 5 min under an atmosphere to dry the sample. Subsequently, the UV lithography template with a channel length of 35–50 µm was designed by KLayout software 5.15.2. By traditional maskless UV lithography technology, an electrode pattern was formed on a photoresist‐coated substrate. Finally, 120‐nm Au/10‐nm Cr films were deposited onto two ends of the nanoribbon to be the electrodes by magnetron sputtering way.

### Photodetection Measurements

The photoresponse characteristics of the (100)‐oriented MoO_2_ nanoribbon were investigated in the self‐built optoelectronic measurement platform, where the photocurrent was automatically recorded by a programmed pico‐ammeter (Keithley Instruments Inc., model 2612B) and a lock‐in amplifier (Sine Scientific Instruments Inc., model OE1201). As schematically shown in Figures 2a and 4a, to effectively avoid the heat transfer in photodetection experiments, the flexible PCB board with a low thermal conductivity of ≈0.20 W m^−1^ K^−1^^[^ ^45^ ^]^ and the PVC board with a low thermal conductivity of ≈0.18 W m^−1^ K^−1^^[^ ^46^ ^]^ were chosen as the sample stage. In addition, the (100)‐oriented MoO_2_ nanodevice was suspended in the measurement chamber to further reduce the heat dissipation. By the above thermal isolation ways, the heat transfer of individual (100)‐oriented MoO_2_ nanodevice could be high‐efficiency isolated under irradiation, leading that it exhibited remarkable self‐powered photoresponse performances. The photoresponse time of the device was measured using a high‐precision oscilloscope (DSOS404A). And the SC450‐4‐PP laser (Fianium, Britain) was used as the excitation source, where the irradiation wavelength was ranging from 0.5 to 1.6 µm. Models LE‐LS‐785 ‐80TSMF laser (Shenzhen Liou Optoelectronics Technology Co., Ltd., China), QCL (Thorlabs, Inc, USA) laser, and Tuotuo laser (Tuotuo Technology Co., Ltd. China) were used as the 0.785‐, 4.7‐, and 10.5‐µm irradiation sources, respectively.

Usually, *I_n_* of Equation (6) in *NEP* calculation is equal to the sum of shot noise (*I*_*sh*._), thermal noise (*I*_*th*._), and 1/*f* noise (*I*_1/*f*_). And the expression of shot noise was written as $I_{sh.}=\sqrt{2qI_{Dark}}$, where *q* is the elementary charge (1.6 × 10^−19^ C) and *I_Dark_* ≈ 10^−11^ A is the dark current of the zero‐biased nanodevice. Thermal noise could be obtained by the equation of $I_{th.}=\frac{4K_{B}T}{R_{d}}\Delta f$, where K_*B*_ is the Boltzmann constant (1.380649 × 10^−23^ J K^−1^), *R_d_* is the device resistance, *T* is the room temperature, andΔ*f*is the chopper frequency. Then, *I*_1/*f*_could be derived from the measurement results of the frequency‐dependent current noise power spectrum (Figures S13,S18, Supporting Information). By this way, *NEP* value of *I*_1/*f*_was found to be approximately equal to that of *I*_*th*._(≈10^−11^ W Hz^−1/2^ @ SiO_2_/Si substrate, ≈10^−12^ W Hz^−1/2^ @ PET substrate). Therefore, the *NEP* value of shot noise *I*_*sh*._  ≈ 10^−14^ W Hz^−1/2^ was far smaller than that of *I*_*th*._, unveiling that the *I*_*sh*._ could be neglected in the experiments, and the measured *NEP* value of individual self‐driven (100)‐MoO_2_ nanoribbons should be highly reliable. This result was also in good agreement with other ohmic‐contact‐type nanodevices.^[^ ^11^, ^33^, ^34^ ^]^

### Computations on the Carrier Mobility and Light Absorbance of MoO_2_ Nanoribbon

All the calculations were carried out in the framework of the DFT with the projector augmented plane‐wave method, as implemented in the Vienna ab initio simulation package.^[^ ^47^ ^]^ The generalized gradient approximation proposed by Perdew–Burke–Ernzerhof was selected for the exchange‐correlation potential.^[^ ^48^ ^]^ The cut‐off energy for the plane wave was set to 500 eV. The energy criterion was kept at 10^−5^ eV in the iterative solution of the Kohn–Sham equation. All the structures were relaxed until the residual forces on the atoms had declined to less than 0.02 eV Å^−1^. To avoid interlaminar interactions, a vacuum spacing of 20 Å was applied perpendicular to the slab.

The carrier mobility could be calculated by the following formula:^[^ ^49^ ^]^

(8) $$\mu_{2D}=\frac{e\hbar^{2}C_{2D}}{K_{B}Tm^{*}m_{d}{\left(E_{1}^{i}\right)}^{2}}$$

where *e* represents the electron charge, *k*_B_ is the Boltzmann constant, and ℏ stands for the simplified Planck constant. The effective mass *m* ^*^ could be expressed as $\frac{1}{m^{*}}=\frac{1}{\hbar }\frac{\partial^{2}E\left(k\right)}{k^{2}}$, where *k* represents the wave vector, *E*(*k*) is the dispersion relation corresponding to *k* near the Fermi level, and the temperature *T* is set at 300 K. The equivalent mass *m_d_* was written as $m_{d}=\sqrt{m_{a}^{*}m_{b}^{*}}$, where $m_{a}^{*}$ and $m_{b}^{*}$ are the effective mass of the crystal along the zigzag and armchair direction, respectively. The elastic modulus *C*_2D_ was given by $C_{2D}=\frac{1}{S_{0}}\frac{\partial^{2}E}{\partial \delta^{2}}$, where *E* means the total energy of the material, *δ* is the strain and *S*_0_ is the surface area of the material. The deformation potential $E_{1}^{i}$ was determined by $E_{1}^{i}=\frac{\Delta V_{i}}{\Delta I/I_{0}}$, where Δ*V_i_* is the energy change of the *i* ^th^ band with compressive and tensile strain, and *I*_0_is the lattice length along the transfer direction and Δ*I*is the variation of *I*_0_ (Δ*I*/*I*_0_ is usually less than 0.5%).

The light absorbance coefficient *α*(*ω*) was obtained by means of the dielectric function using the formula:

(9) $$\alpha \left(\omega \right)=\sqrt{2}\omega {\left[\sqrt{\varepsilon_{1}^{2}\left(\omega \right)+\varepsilon_{2}^{2}\left(\omega \right)}\;-\;\varepsilon_{1}\left(\omega \right)\right]}^{\frac{1}{2}}$$

where *c* is the speed of light, ε_1_(ω) and ε_2_(ω) are the real and imaginary parts of the dielectric functions, respectively.

### Theoretical Calculation on the Electrical Conductivity of Individual MoO_2_ Nanoribbon

The DFT calculations were done with the projector augmented plane‐wave basis, which was implemented in the Vienna ab‐initio simulation package.^[^ ^47^ ^]^ And the plane‐waves were cut‐off at 550 eV. The exchange‐correlations of electrons were described by the generalized gradient approximations with the form proposed by Perdew, Burke, and Ernzerhof.^[^ ^48^ ^]^ The energy converge criterion for solving self‐consistent Kohn–Sham equations was 10^−7^ eV. All the structures in this study were fully relaxed until the Hellman–Feynman smaller than 0.05 eV Å^−1^. To obtain the self‐consistent charge density, the Brillouin zone (BZ) was sampled with 11 × 13 × 11, using the scheme of Monkhorst–Pack.^[^ ^50^ ^]^ Then, a much denser grid 57 × 57 × 7 was used to interpolate BZ to obtain transport properties. The scattering effects were approached by considering effective electron–phonon couplings via the acoustic deformation potential model, as implemented in AMSET.^[^ ^51^ ^]^ Then, the electrical transport properties were computed through the linearized Boltzmann equation.^[^ ^52^ ^]^ First, the spectral conductivity (Σ) was calculated:

(10) $$\sum \left(\varepsilon \right)=\sum_{n}\int_{BZ}\frac{dk}{{\left(2\pi \right)}^{3}}v_{nk}⊗v_{nk}\tau_{nk}\delta \left(\varepsilon \;-\;\varepsilon_{nk}\right)$$

where *v_nk_* is the velocity of the band electron, ε_*nk*_ is the eigen‐energy of the band electron, and τ_*nk*_ is the relaxation time deduced from the scattering. The electrical conductivity (σ) was obtained by integrating:

(11) $$\sigma =e^{2}\int \sum \left(\varepsilon \right)\left(-\frac{\partial f^{0}\left(\varepsilon \right)}{\partial \varepsilon }\right)d\varepsilon$$

(12) $$f^{0}=\frac{1}{\exp\left[\left(\varepsilon \;-\;E_{f}\right)/K_{B}T\right]+1}$$

where *f* ^0^, K_*B*_, and *E_f_* are the Fermi–Dirac distribution at given temperature (*T*), Boltzmann constant, and Fermi level, respectively.

### Theoretical Calculation on the Thermal Conductance and Seebeck Coefficient of the MoO_2_ Nanoribbon

The DFT calculations were done with the projector augmented plane‐wave basis, which was implemented in the Vienna ab‐initio simulation package.^[^ ^47^ ^]^ And the plane‐waves were cut‐off at 550 eV. The exchange‐correlations of electrons were described by the generalized gradient approximations with the form proposed by Perdew–Burke–Ernzerhof.^[^ ^48^ ^]^ The energy converge criterion for solving self‐consistent Kohn–Sham equations was 10^−7^ eV. The Brillouin zone was sampled with ultra‐high resolution better than 0.01 Å^−1^, using the scheme of Monkhorst–Pack.^[^ ^50^ ^]^ All the structures in this study were fully relaxed until the Hellman–Feynman smaller than 0.05 eV Å^−1^. The thermal conductance and Seebeck coefficient were estimated by solving the linearized Boltzmann equation with constant relaxation time approximation (RTA), as implemented in the Boltztrap2 code.^[^ ^52^ ^]^ The relaxation time was set to ≈0.3 ps, as deduced by the Drude conductance and experimental measurements.

### Calculation on the Thermoelectric Voltage on an Individual MoO_2_ Nanodevice

The numerical simulation was implemented by COMSOL Multiphysics 6.1. The simulation model contained three different materials: SiO_2_/Si with a thermal conductivity of 100 W m^−1 ^K^−1^,^[^ ^31^ ^]^ PET with a thermal conductivity of 0.04 W m^−1 ^K^−1^,^[^ ^35^ ^]^ and the MoO_2_ nanoribbon with a thermal conductivity of ≈400 W m^−1 ^K^−1^ (Figure S25, Supporting Information). During the calculation process, one end of the substrate was adiabatic, while the other end had a continuous heat flux input, resulting in a certain temperature difference between these two ends. In this situation, the thermoelectric potential was generated.

The distribution of the temperature field was governed by heat conduction and heat convection, which could be expressed by:^[^ ^53^ ^]^

(13) $$Q=u_{t}\cdot \nabla T+\nabla \;\cdot \;q$$

(14) $$q=K_{T}\nabla T$$

where ***q*** is the heat flux, *u_t_* is the fluid velocity (*µ_t _* = 0 in this case), *T* is the temperature of the materials, and *K*_T_ is the thermal conductivity of the materials, *Q* is the total thermal energy. The equation of ***q*** could be rewritten by calculating the thermoelectric effect:^[^ ^53^ ^]^

(15) q=−κ∇T+PJ

(16) $$J=-\sigma \left(\nabla V+S\nabla T\right)$$

where κ, *P*, ***J***, S, and V are the thermal conductivity, Peltier coefficient, current density, Seebeck coefficient, and thermoelectric potential, respectively. By combination of the above equations and the boundary conditions, which were defined by the geometric profile of the materials, the nanoribbon temperature and the thermoelectric potential distribution along the nanoribbon axis could be solved, respectively.

## Conflict of Interest

The authors declare no conflict of interest.

## Supporting information



Supporting Information

## Data Availability

The data that support the findings of this study are available from the corresponding author upon reasonable request.
