# Supplementary material for: Facet‐Engineered (100)‐Oriented MoO2 Nanoribbons for Broadband Self‐Powered Photodetection
Source: Adv Sci (Weinh). 2025 Aug 25;12(43):e10753. doi: 10.1002/advs.202510753 (PMC12631821; doi:10.1002/advs.202510753)
Supplement: Supplementary file 1 — Supporting Information [file ADVS-12-e10753-s001.docx]

***Supporting Information***

**Facet-Engineered (100)-Oriented MoO_2_ Nanoribbons for Broadband Self-Powered Photodetection**

*Haojian Lin, Ximiao Wang, Tianrong Yi, Jidong Liu, Jiahao Wu, Shaojing Liu, Yang Chai, Fei Liu*, Di Wu*, Huanjun Chen*, and Wenjing Zhang**


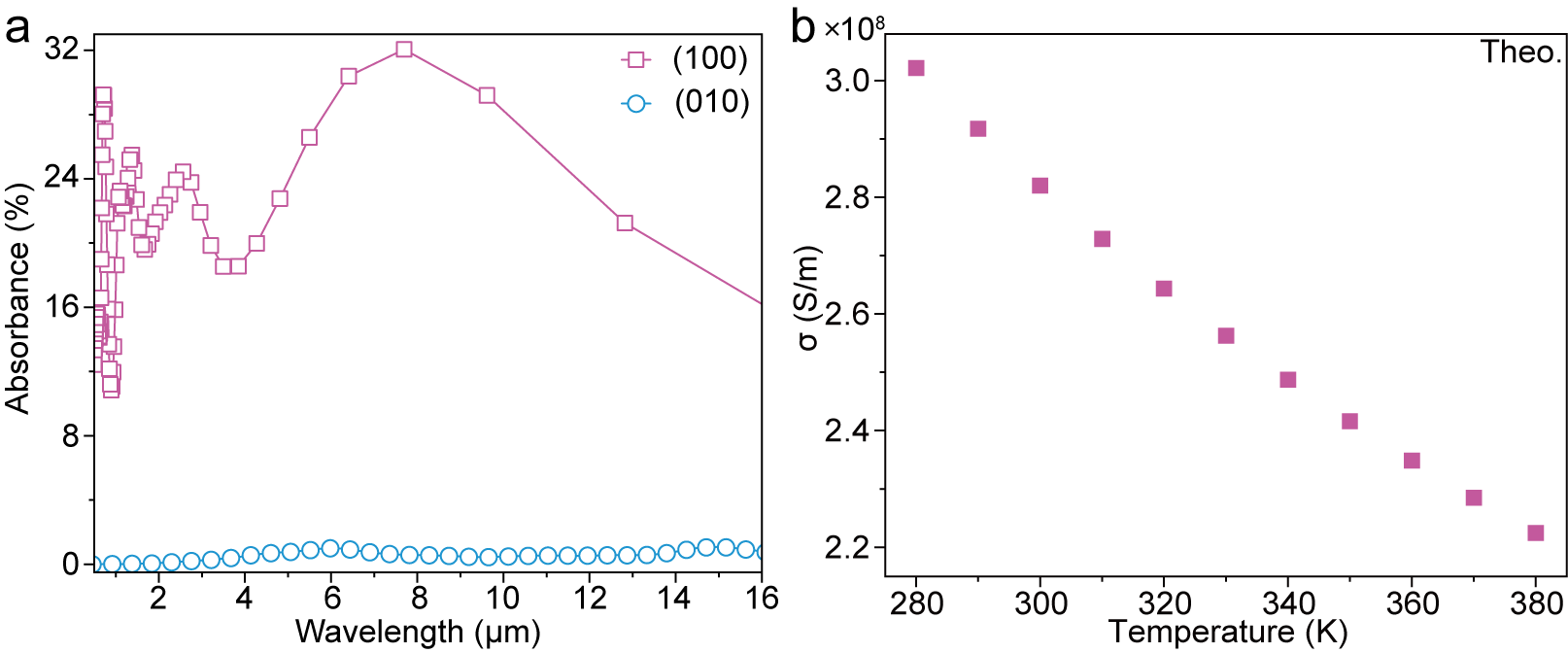


**Figure S1.** a) Theoretical light absorbance of (100)- and (010)-oriented MoO_2_ nanostructures in a very wide spectral range from 0.5 to 16 μm. b) Theoretical electrical conductivity of (100)-oriented MoO_2_ nanostructure at different temperatures.


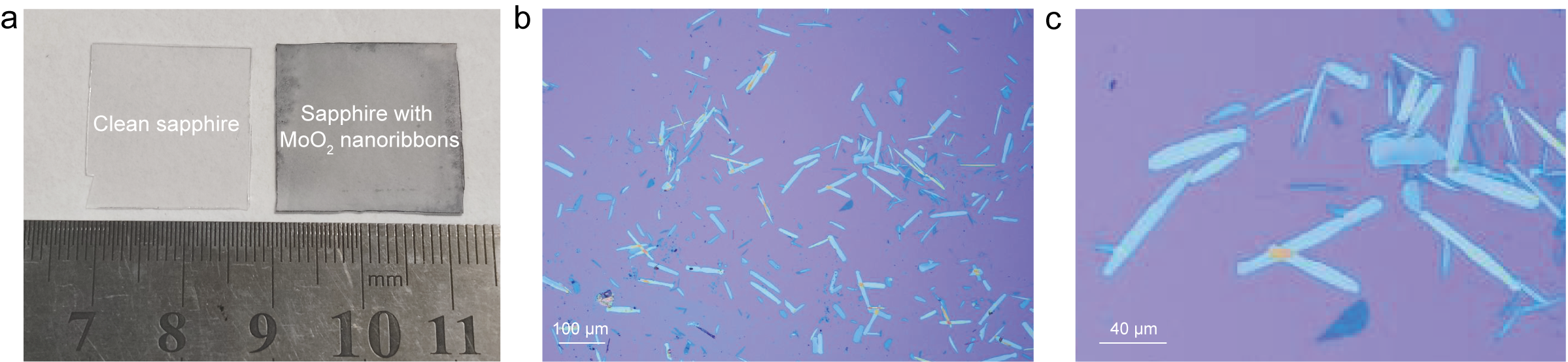


**Figure S2.** a) Photograph of the as-synthesized product on c-sapphire substrate. b, c) Low- and high-magnification optical microscope images of the transferred (100)-oriented MoO₂ nanoribbons on SiO₂/Si substrate.


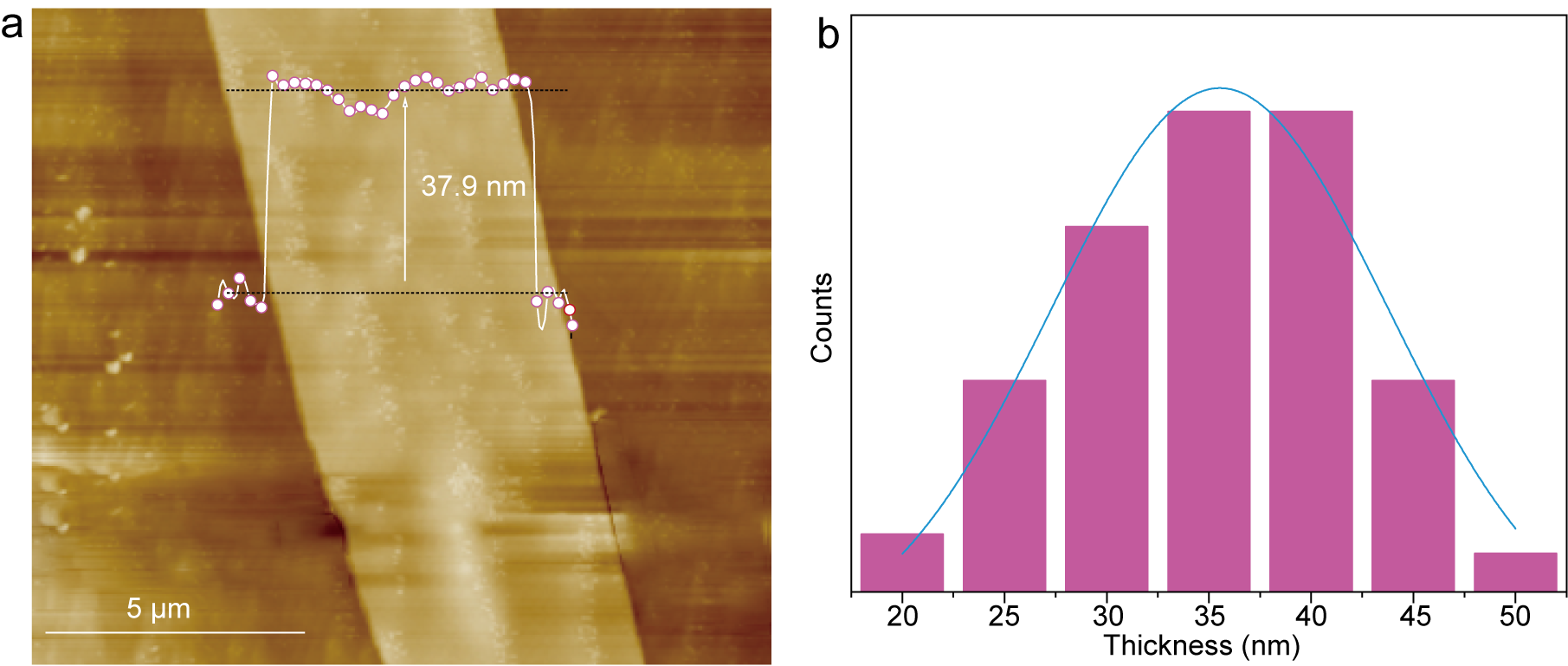


**Figure S3.** a) Representative AFM topographical image of a (100)-oriented MoO_2_ nanoribbon. b) The statistical thickness distribution of the MoO_2_ nanoribbons by APCVD method


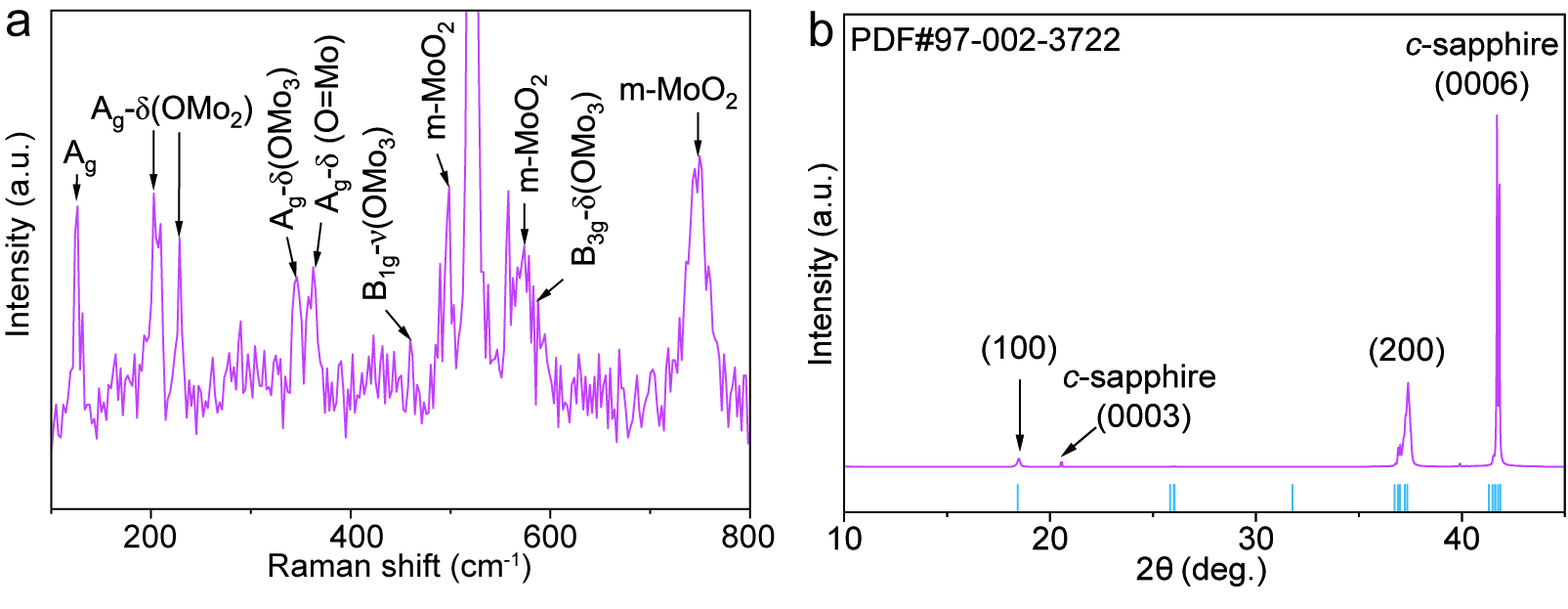


**Figure S4.** Raman spectroscopy (a) and X-ray diffraction (XRD) pattern (b) of the as-grown (100)-oriented MoO_2_ nanoribbons.


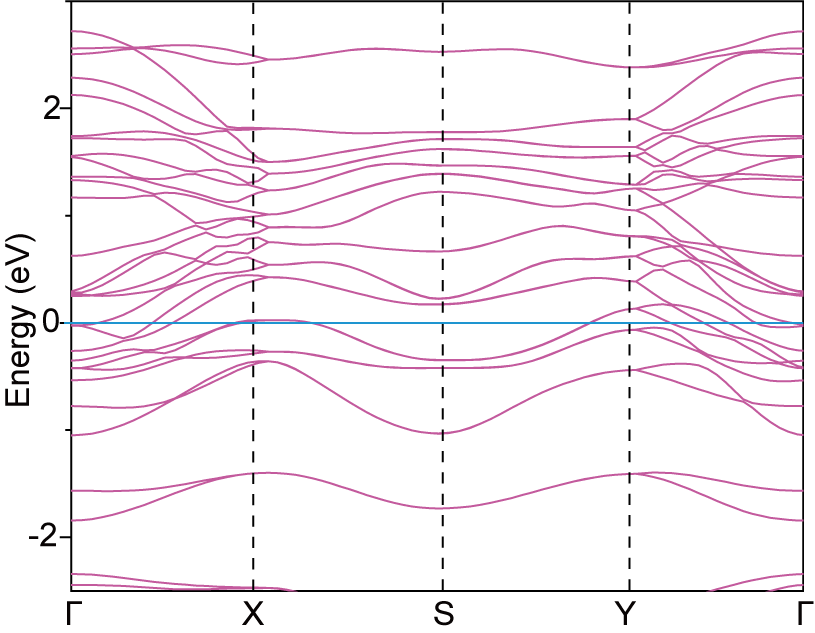


**Figure S5.** Band structure of individual (100)-oriented MoO_2_ nanoribbon by DFT calculation.


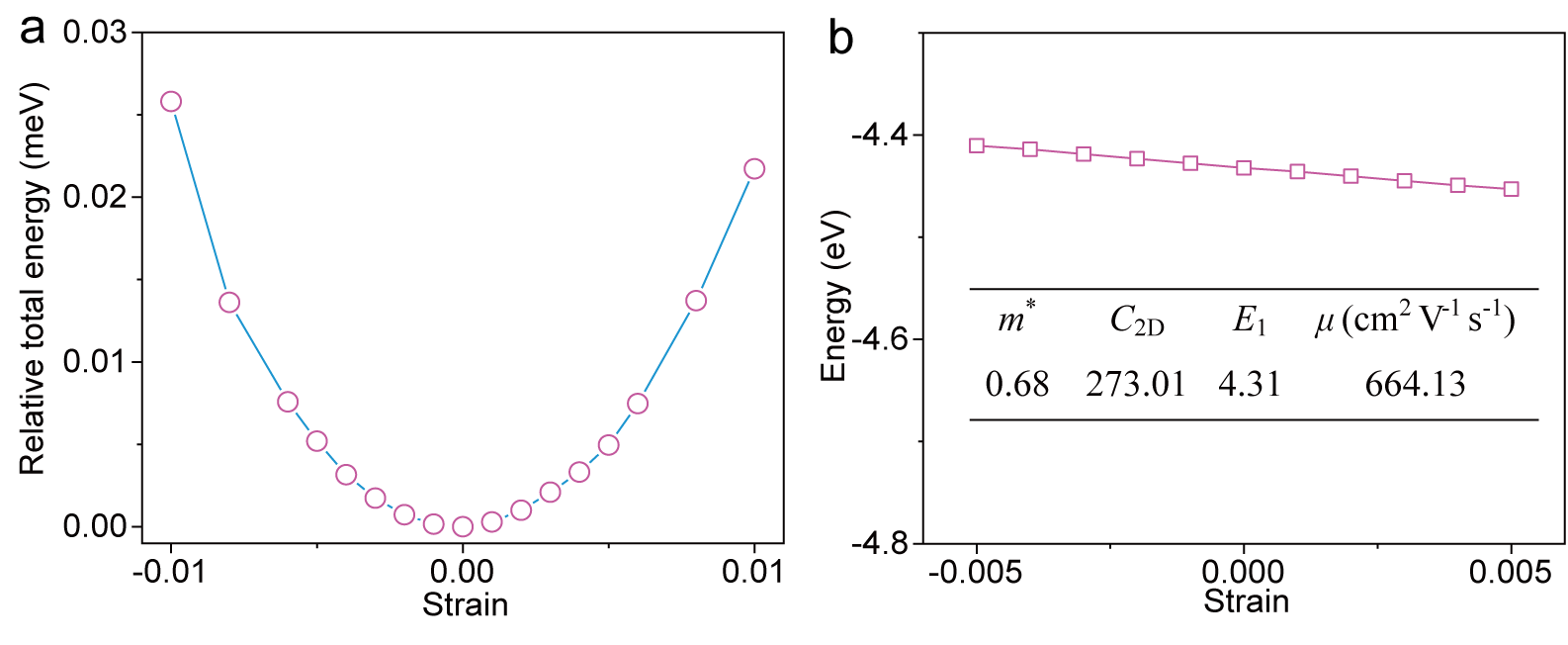


**Figure S6.** a) The energy-strain relationship of (100)-oriented MoO_2_. b) The shift of electron energy for (100)-oriented MoO_2_ with respect to the vacuum energy as a function of the transverse strain. And the inset gives the corresponding electron mobility under different strains.


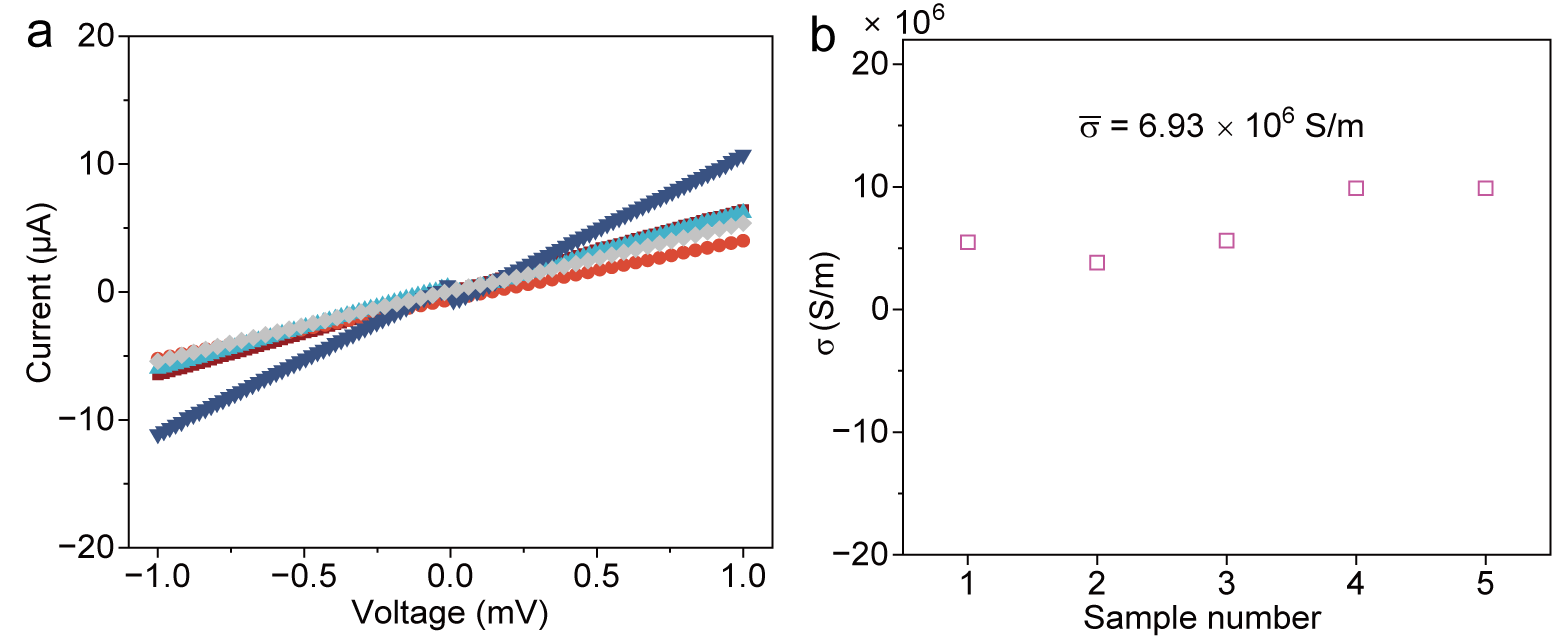


**Figure S7.** a) Representative current-voltage characteristics of individual (100)-oriented MoO_2_ nanoribbons. b) Their corresponding electrical conductivity distribution curve.

In this work, two types of substrates, SiO_2_/Si and c-sapphire, were used to synthesize MoO_2_ nanoribbon with different base planes, respectively. Because the MoO_2_ (001) plane (a = 0.56 nm, b = 0.48 nm) has a nice lattice match with c-sapphire (0001) plane (a = b = 0.475 nm),^[1]^ the evaporated MoO_2_ molecules will firstly tend to epitaxially grow on the c-sapphire substrate, and act as the seeds for the foreign MoO_2_ molecules. With the progression of the reaction, the growth speed of the (100) facet of the seeds is gradually predominant over the (001) facet according to the surface-confined successive growth model, leading to the formation (100)-orient MoO_2_ nanoribbon.^[2]^ This mechanism is also proved by the inset cross-sectional image of Figure 1c and HRTEM (Figure 1e) result. But when changing the growth substrate from c-sapphire substrate to SiO_2_/Si substrate, the MoO_2_ (010) plane with the highest surface Gibbs free energy tends to be the growth plane with the highest nucleating rate due to the existence of massive dangling bonds on amorphous SiO_2_ layer,^[1a, 3]^ inducing the formation of the (010)-oriented MoO_2_ nanoribbon. Therefore, (010)-oriented and (100)-oriented MoO_2_ nanoribbons can be controllably prepared by choosing different substrates.

From Figure S8a-c, it can be observed that the unit cells of (100)-oriented MoO₂ are arranged in a rectangular pattern, and the spacings between adjacent lattices are ~0.28 nm and ~0.48 nm, respectively. For the (010)-oriented MoO₂, the surface atoms are combined in the form of a regular hexagonal unit cell with a side length of ~0.33 nm, as shown in Figure S8d-f. In addition, the atomic arrangement symmetry of the (100) plane is clearly lower than that of the (010) plane, which should be responsible for the excellent polarization-sensitive photodetection performances of the (100)-oriented MoO₂ nanoribbons.


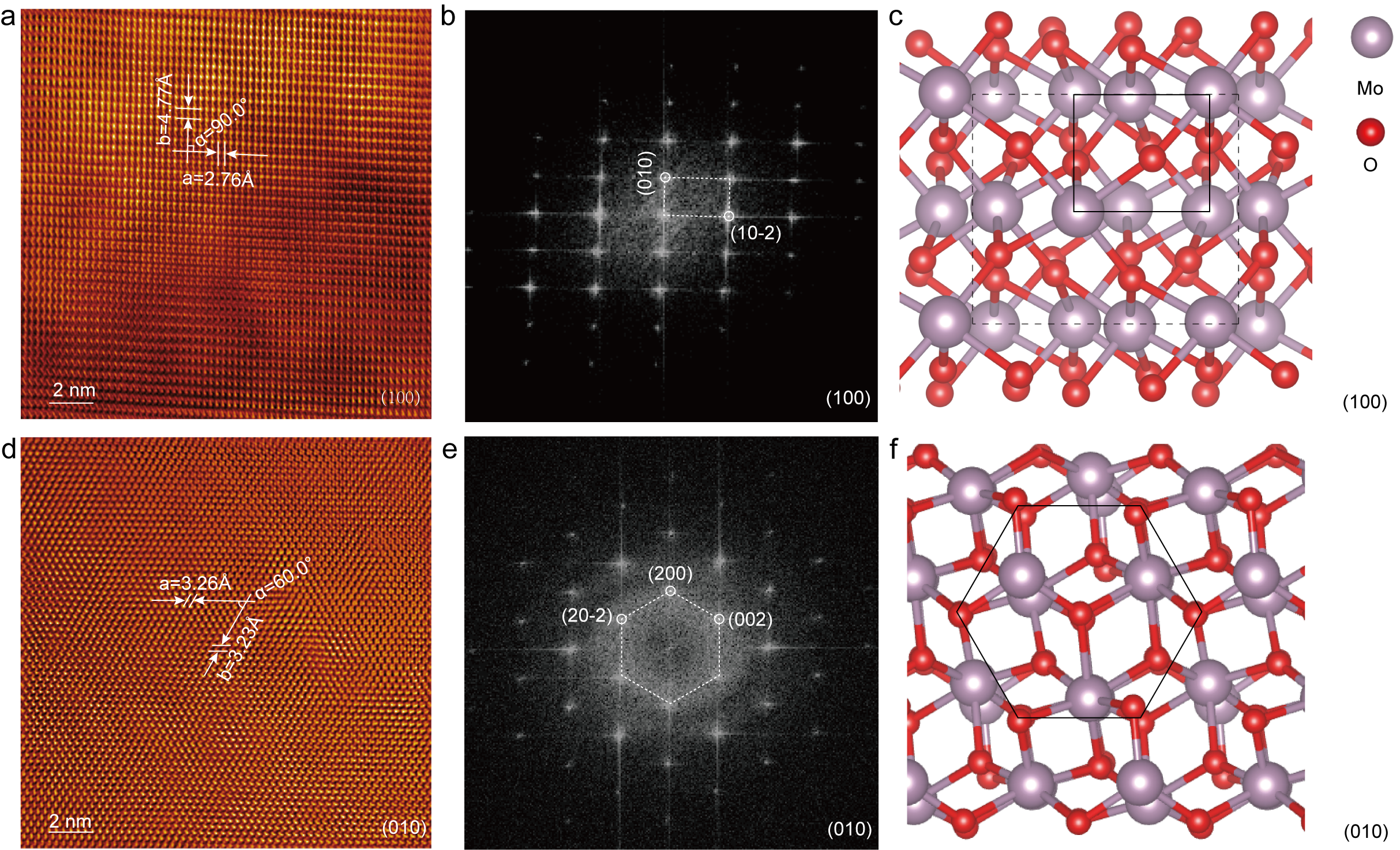


**Figure S8**. a, d) Typical HRTEM images of the (100)- and (010)-oriented MoO_2_ nanoribbons, respectively. b, e) Their corresponding FFT patterns. c, f) Top-view of the rectangular and hexagonal unit cells of the (100)- and (010)-oriented MoO_2_ nanoribbon by DFT calculation, respectively.


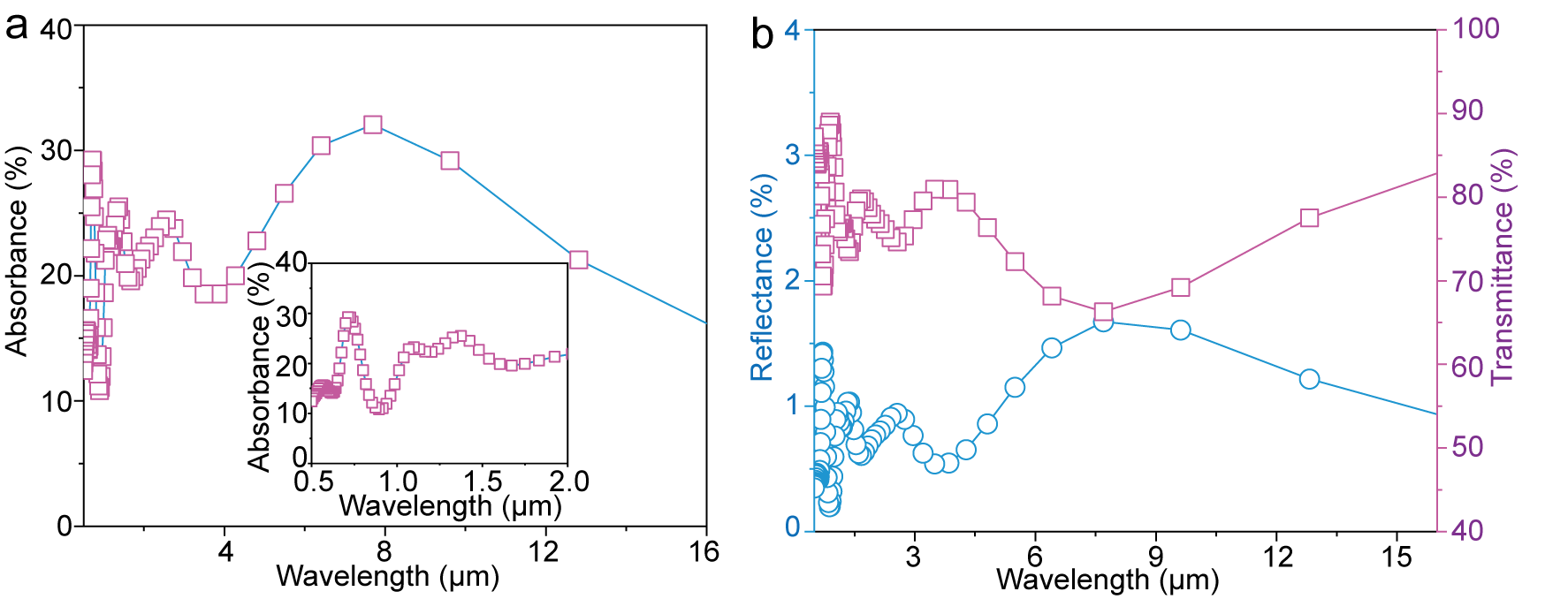


**Figure S9.** Theoretical light absorbance, reflectance and transmittance of the (100)-oriented MoO_2_ nanoribbons in the wavelength range from 0.5 to 16 μm.


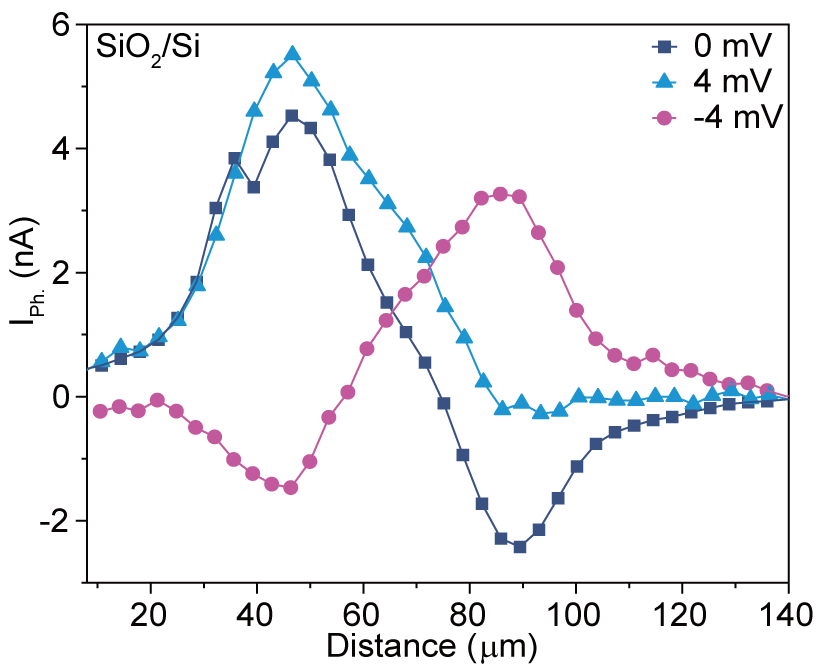


**Figure S10.** Photocurrent profiles of the (100)-oriented MoO_2_ nanoribbon photodetector on SiO_2_/Si substrate, where the applied voltage adopts 0, +4 and -4 mV under 10.5-μm irradiation, respectively. The distance stands for the length from the irradiation end to a given site along the nanoribbon axis.


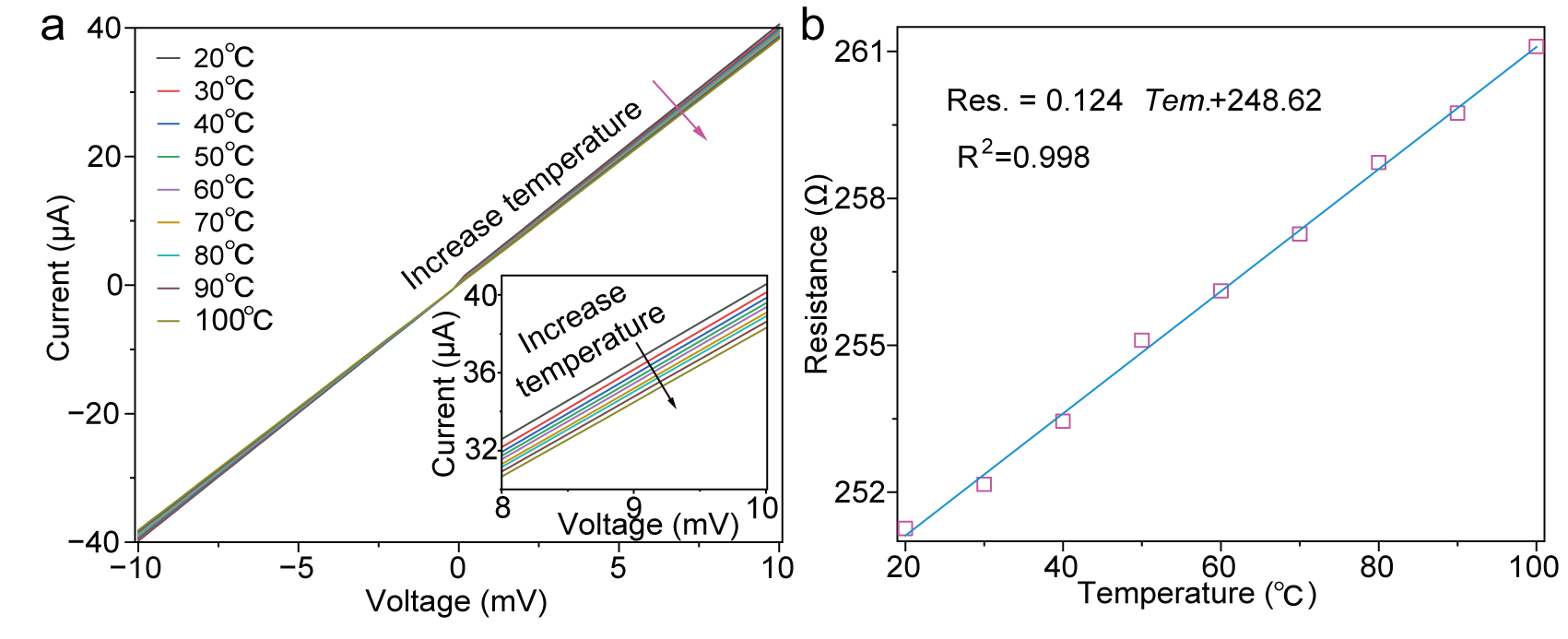


**Figure S11.** a) Typical I-V curves of individual (100)-oriented MoO₂ nanoribbon at different temperatures. b) Their corresponding electrical resistance-temperature curve.


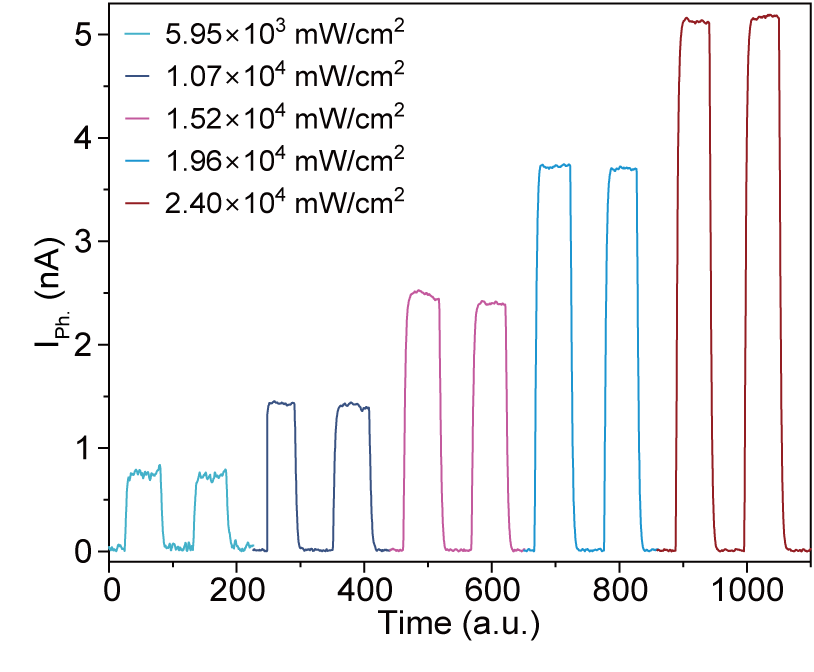


**Figure S12.** The on-off behaviors of the zero-biased (100)-oriented MoO_2_ photodetector on SiO_2_/Si substrate under 10.5-μm irradiation with different light power densities.


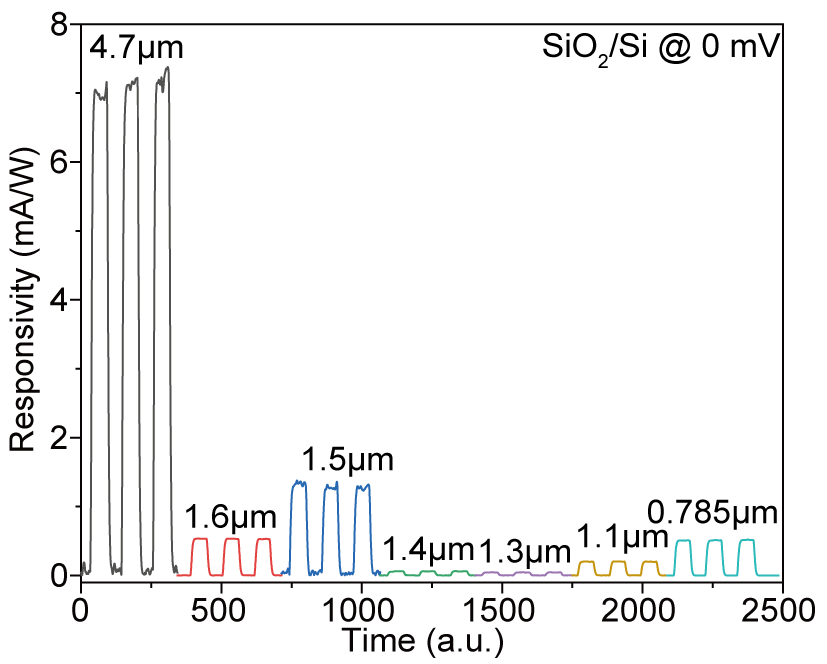


**Figure S13.** Photoresponsivity performances of the zero-biased (100)-oriented MoO_2_ photodetector on Si/SiO_2_ substrate under 4.7-, 1.6-, 1.5-, 1.4-, 1.3-, 1.1-, 0.785-μm excitations, in which the light power density is 2.52×10^4^, 7.03×10^5^, 5.07×10^4^, 7.63×10^5^, 7.31×10^5^, 1.22×10^6^ and 2.25×10^5^ mW/cm^2^, respectively.


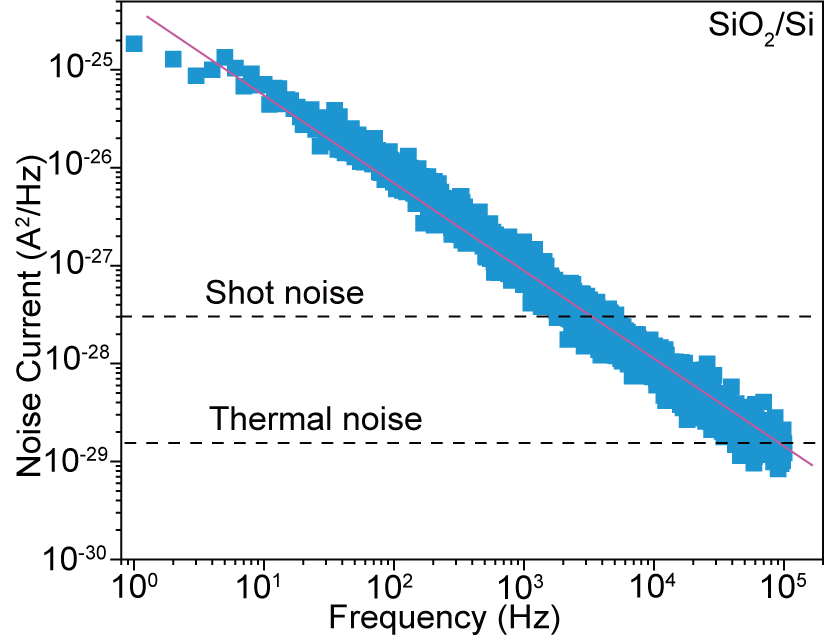


**Figure S14**. The frequency-dependent current noise power spectrum of the zero-biased (100)-oriented MoO_2_ photodetector on SiO_2_/Si substrate.


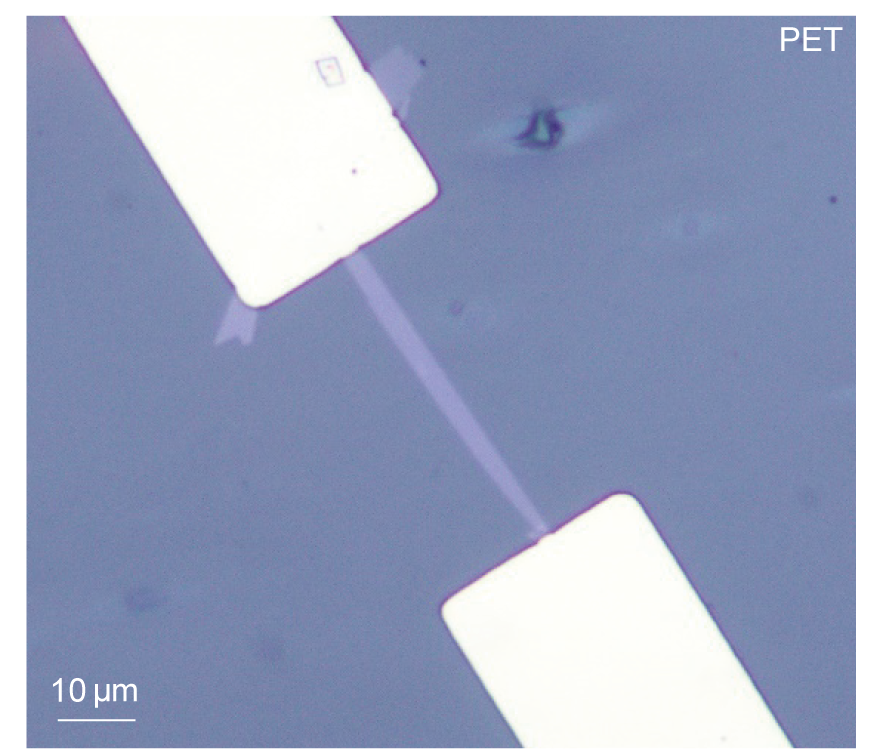


**Figure S15**. Optical microscope image of individual (100)-oriented MoO_2_ photodetector on PET substrate.


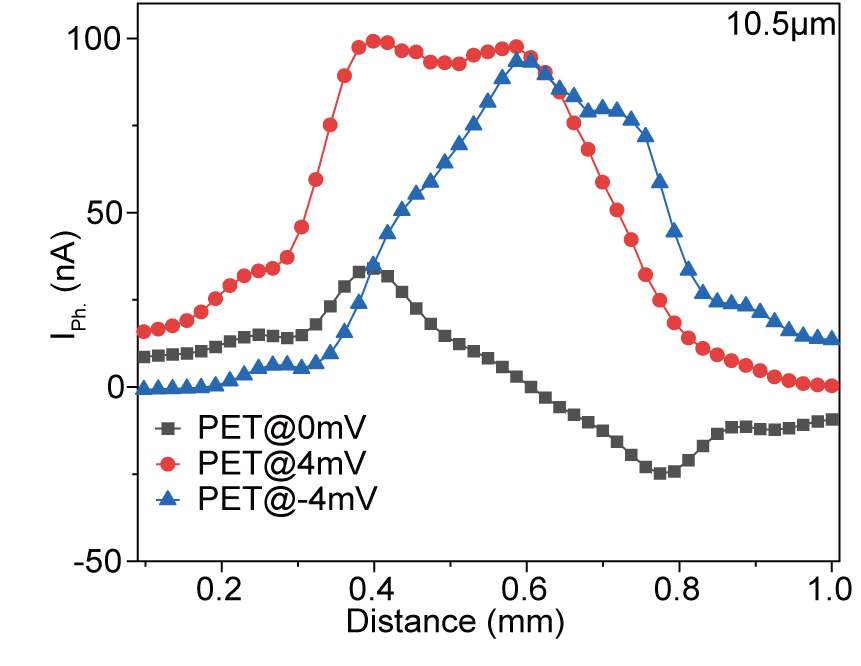


**Figure S16**. Photocurrent-distance curves of the (100)-oriented MoO_2_ photodetector on PET substrate, where the applied voltage is 0, +4 and -4 mV and the light power density is 2.40×10^4^ mW/cm^2^.


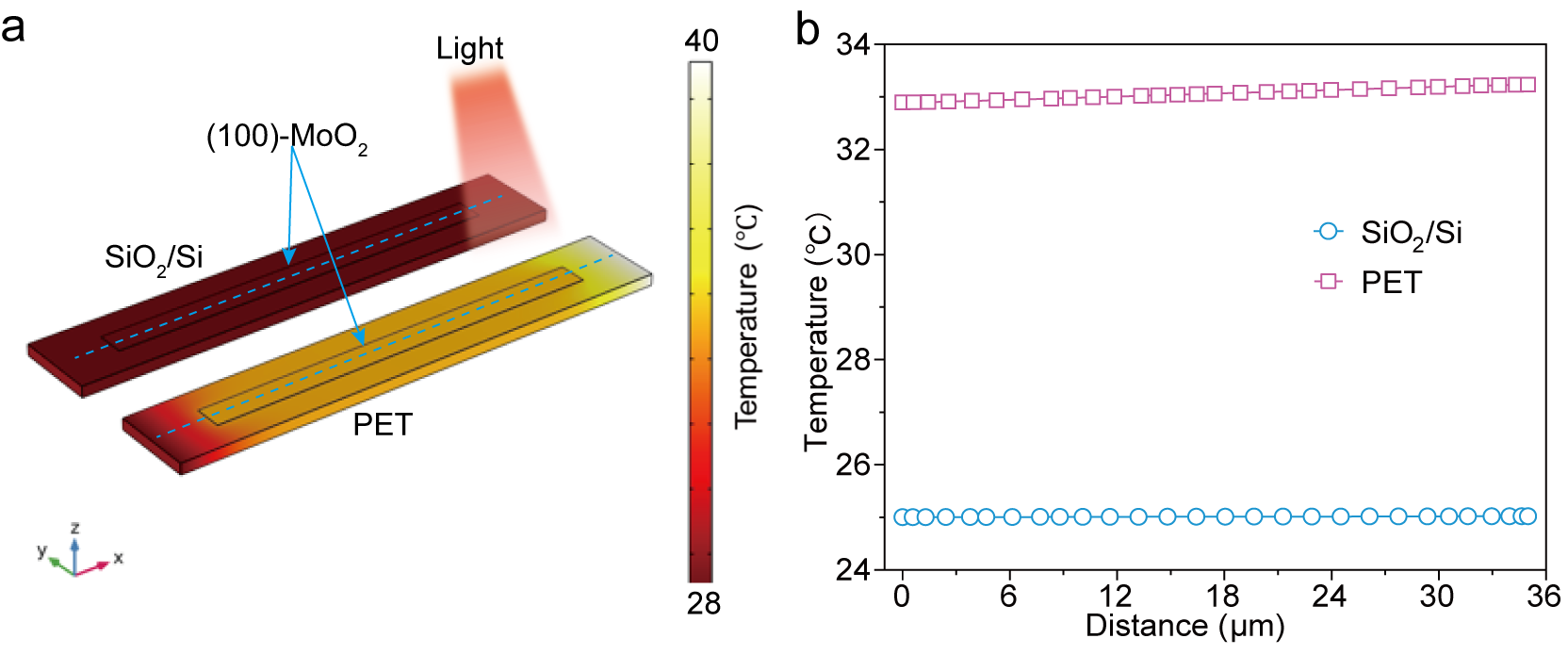


**Figure S17.** The simulating temperature distribution image (a) and curve (b) of individual (100)-oriented MoO₂ nanoribbon under the same 10.5-μm irradiation on SiO₂/Si and PET substrates, respectively.


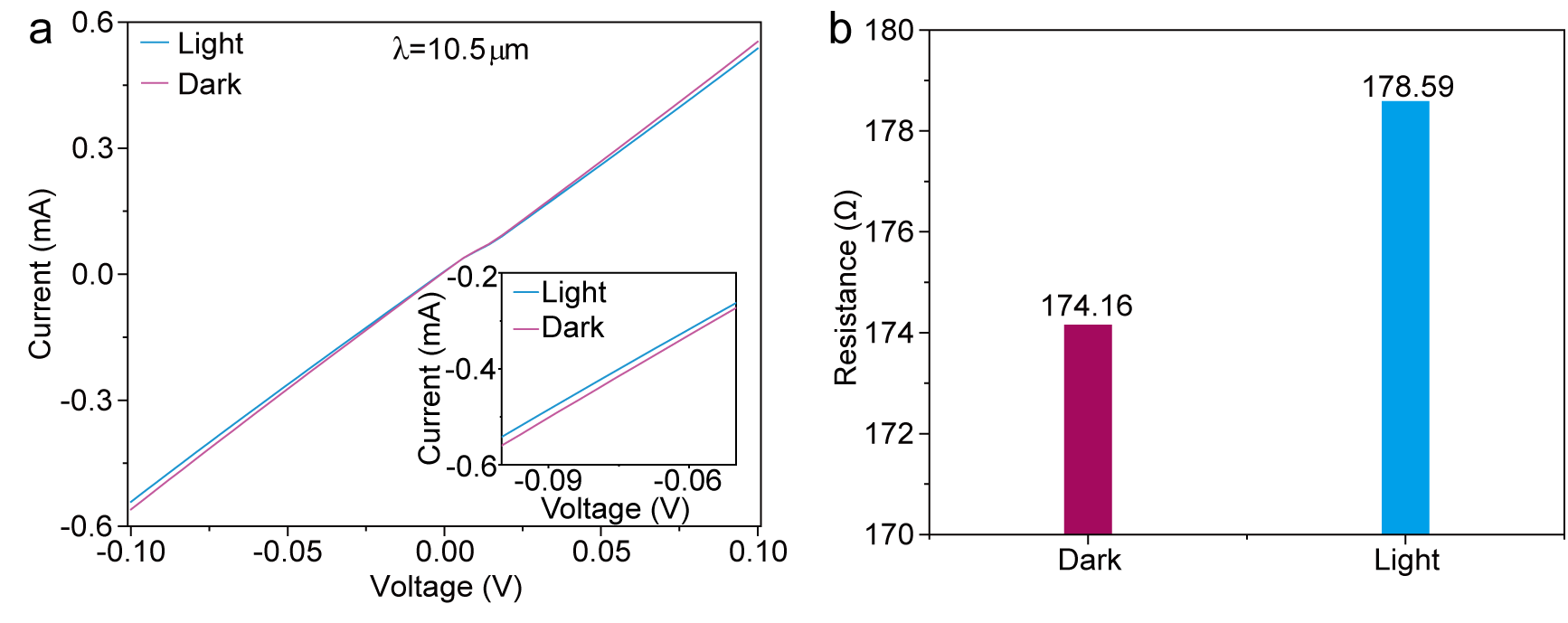


**Figure S18.** a) Typical I-V curves of the photodetector fabricated on a PET substrate under dark and illuminated conditions. The 10.5-μm irradiation has a light power density of 2.40 × 10⁴ mW/cm². b) The calculated electrical resistance of individual nanoribbon under dark and light conditions based on the data from (a).


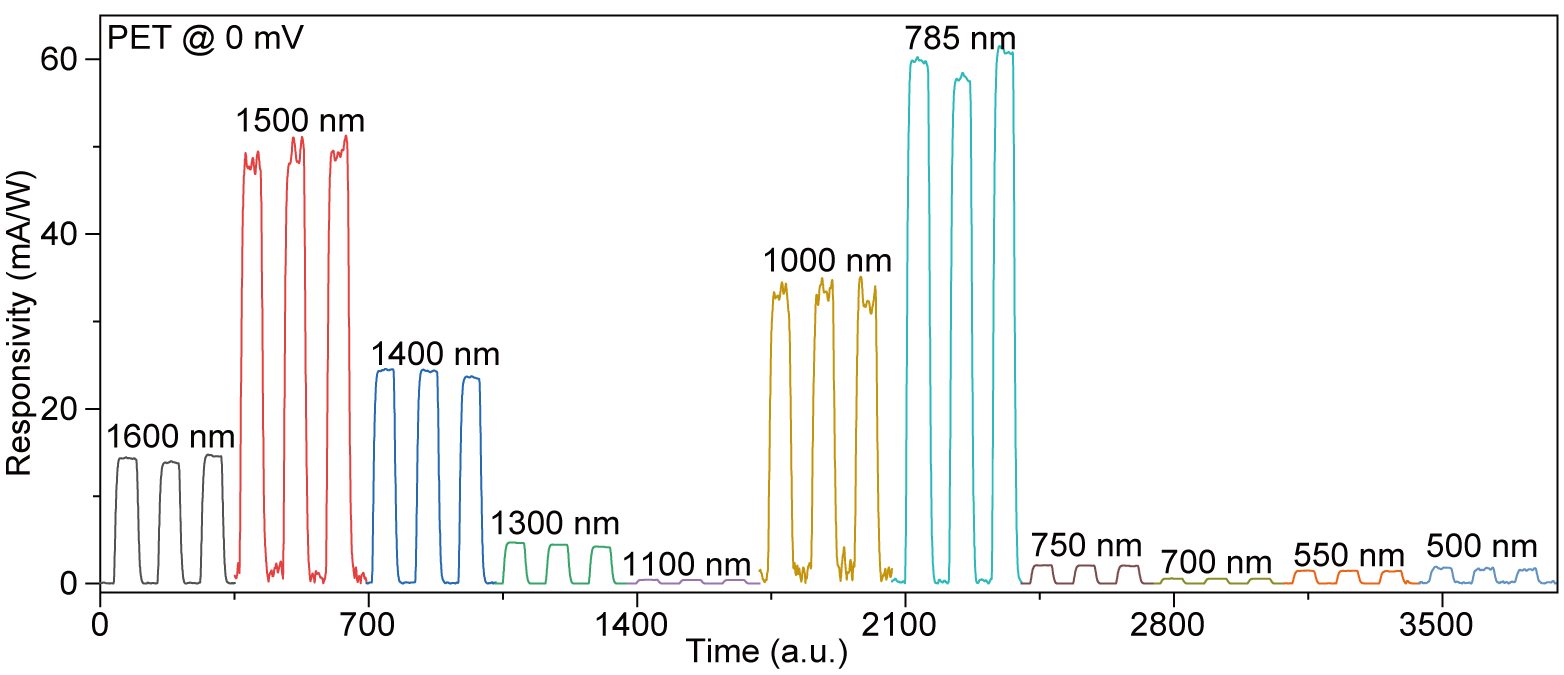


**Figure S19.** Photoresponsivity performances of the zero-biased (100)-oriented MoO_2_ photodetector on PET substrate under 1.6-, 1.5-, 1.4-, 1.3-, 1.1-, 1-, 0.785-, 0.75-, 0.7-, 0.55- and 0.5-μm irradiations, in which the light power density is 7.03×10^5^, 5.07×10^4^, 7.63×10^5^, 7.31×10^5^, 1.22×10^6^, 7.55×10^4^, 2.25×10^5^, 6.88×10^4^, 3.08×10^5^, 2.19×10^5^ and 2.07×10^4^ mW/cm^2^, respectively.


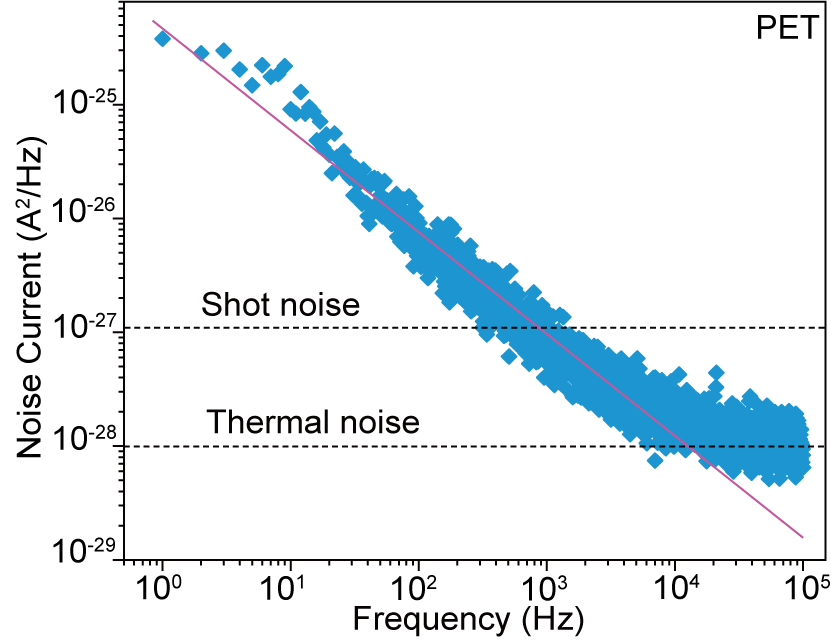


**Figure S20**. The frequency-dependent current noise power spectrum of the zero-biased (100)-oriented MoO_2_ photodetector on PET.


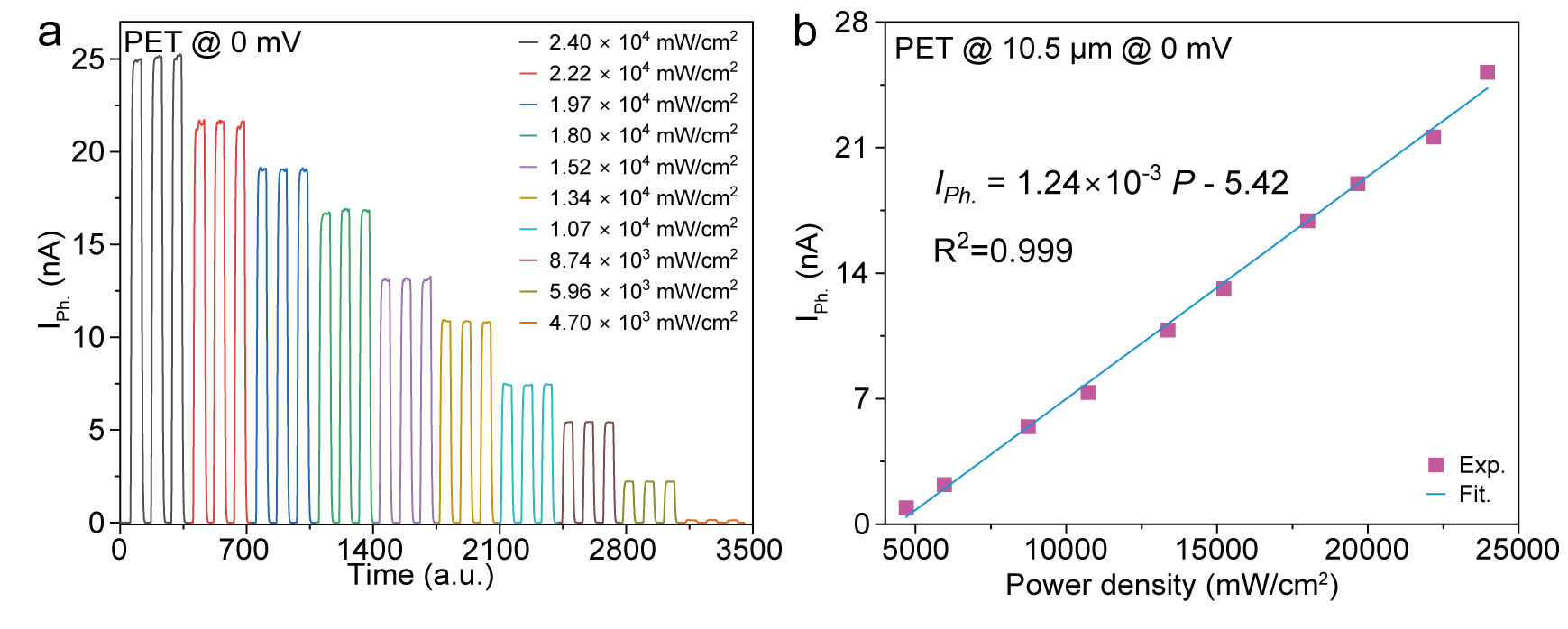


**Figure S21.** a) The light power density-dependent photocurrent curves of the zero-biased (100)-oriented MoO_2_ photodetector on PET substrate under 10.5-μm irradiation. b) Their corresponding power density-dependent photocurrent curve.


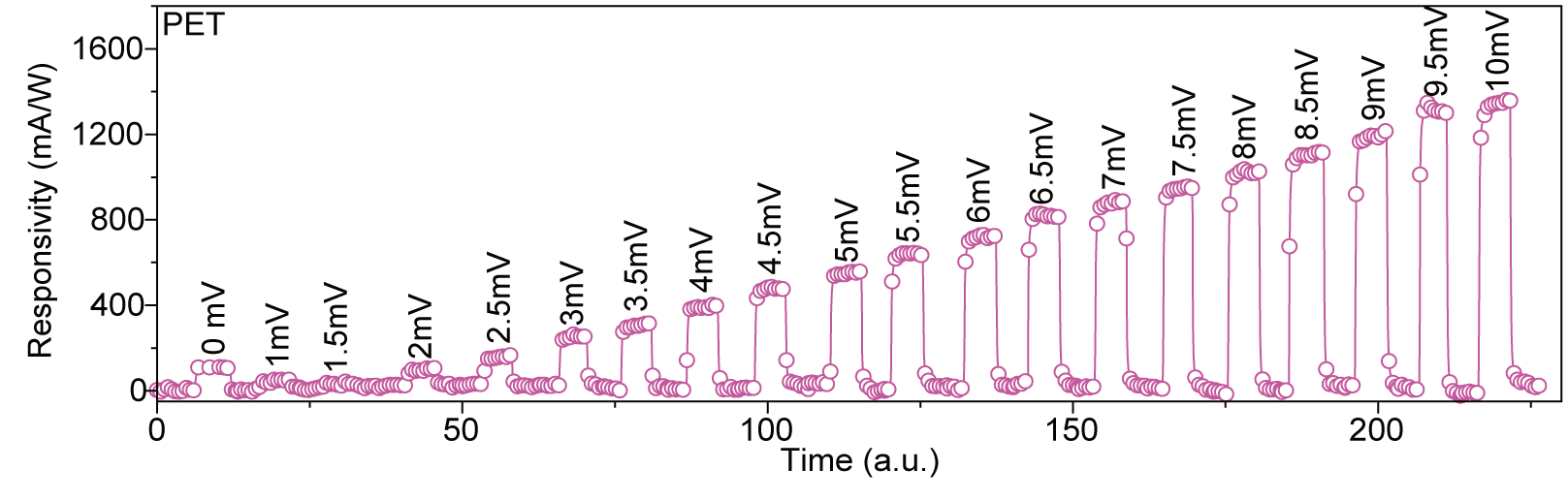


**Figure S22.** Photoresponsivity performances of individual (100)-oriented MoO_2_ nanoribbon photodetector on PET substrate under 10.5-μm irradiation, where V_DS_ ranges from 0 to +10 mV.


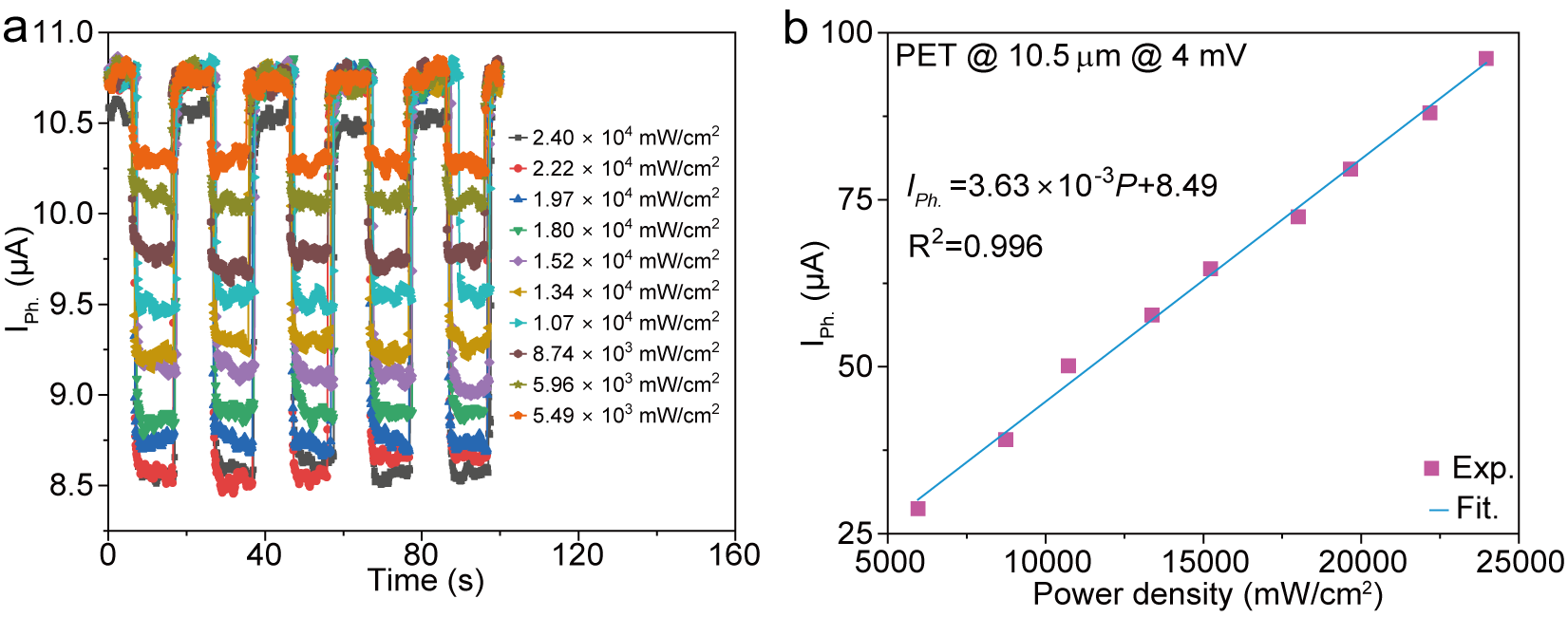


**Figure S23.** a) Photocurrent curves of the +4 mV-biased flexible (100)-oriented MoO₂ photodetector under 10.5-μm irradiation as a function of the light power density. b) Their corresponding photocurrent to the light power density curve.


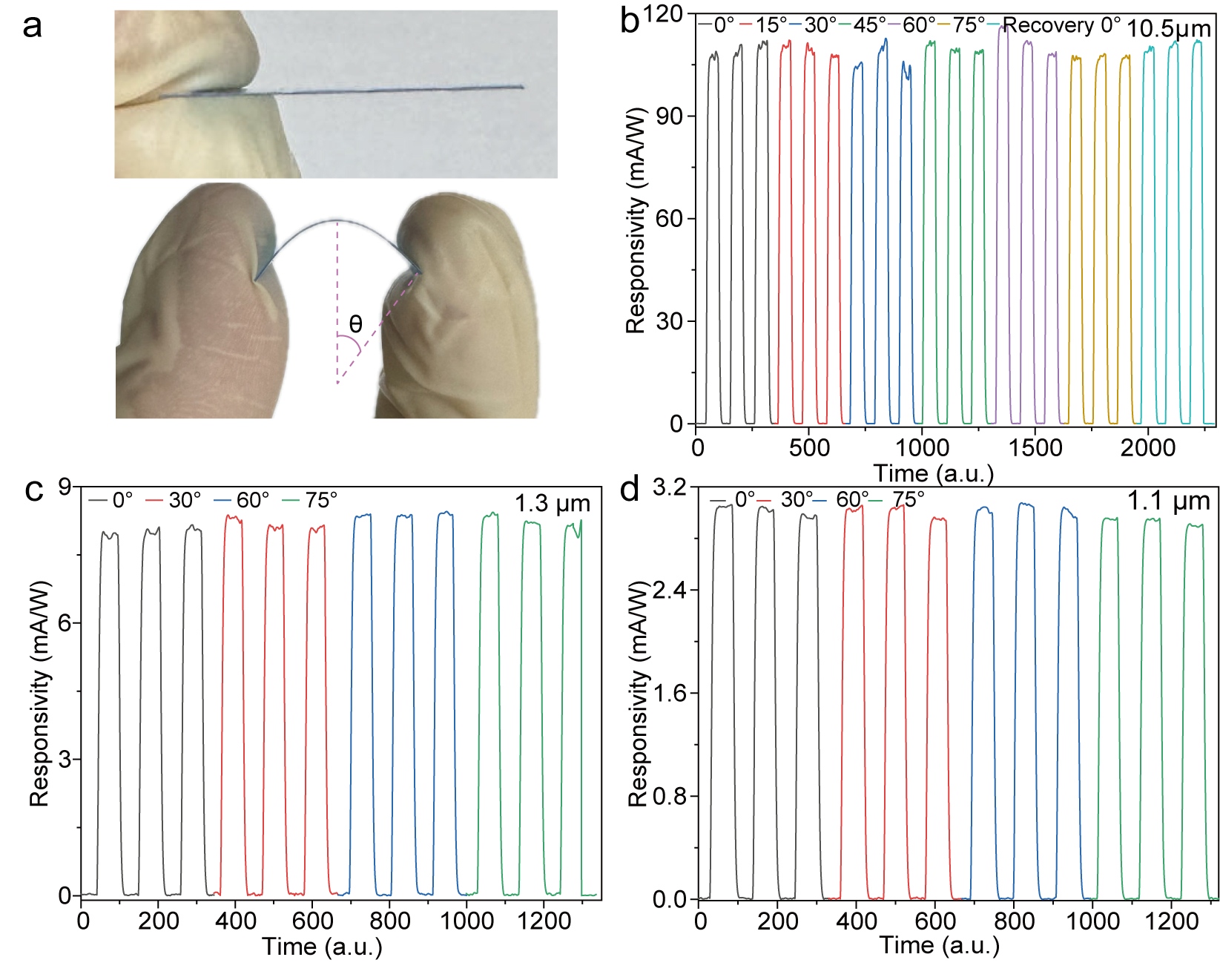


**Figure S24.** Self-powered photoresponse performances of the flexible (100)-oriented MoO_2_ photodetector in a broadband spectral range. a) Photographs of the zero-biased flexible photodetector with and without deformation. b, c, d) The broadband photoresponse performances of the self-powered flexible (100)-oriented MoO_2_ photodetector under different bending angles, in which the light power density of 2.40×10^4^, 7.31×10^5^, 1.22×10^6^ mW/cm^2^ are adopted for 10.5-μm, 1.3- and 1.1-μm irradiations, respectively.


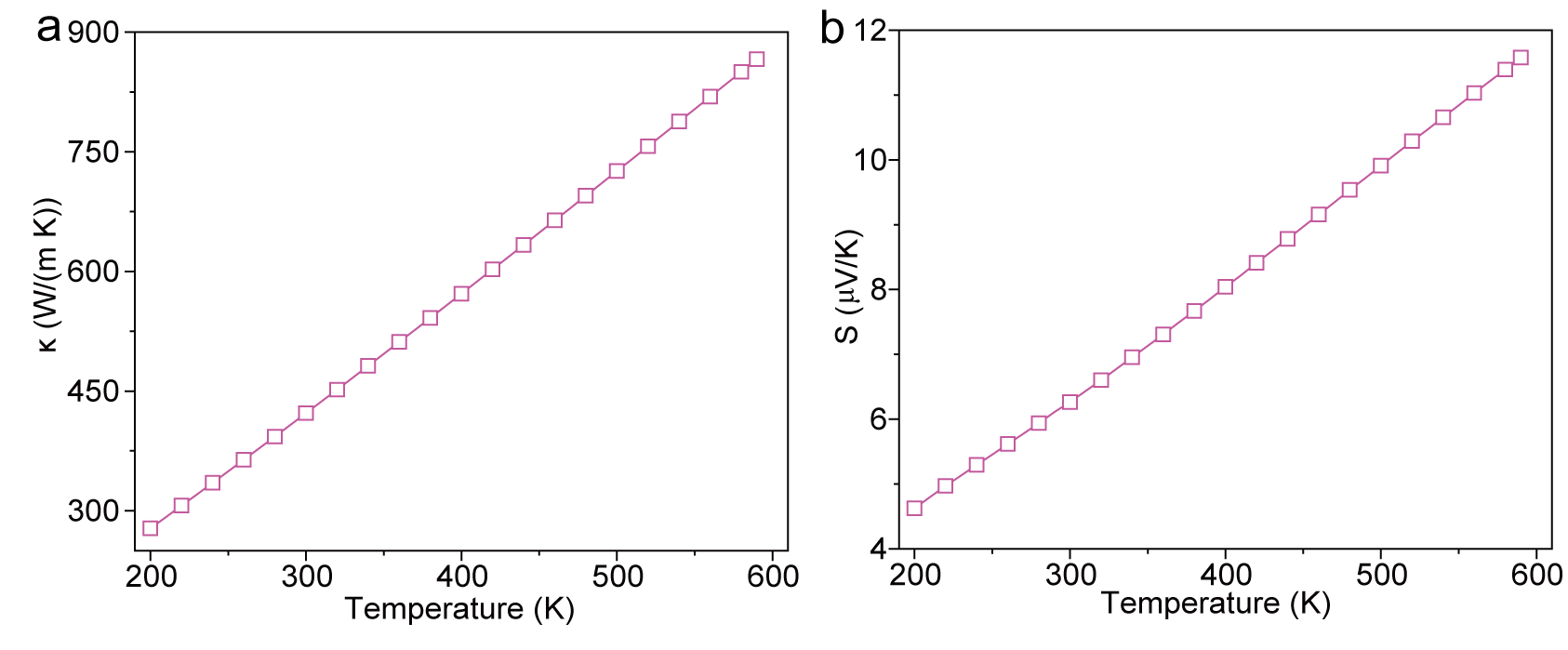


**Figure S25.** Theoretical thermal conductivity (a) and Seebeck coefficient (b) of (100)-oriented MoO_2_ nanoribbons at various temperatures.

**Table S1.** Comparison of the photoresponse performances of broadband self-powered nanoscale photodetectors on rigid substrate at room temperature.

| **Materials**  **(mechanism)** | **Range**  **(μm)** | ***R_Ph._***  **(mA/W)** | ***t_on_*/*t_off_***  **(μs)** | ***NEP***  **(****W/Hz^0.5^)** | ***D****  **(Jones)** | **Ref.** |
| --- | --- | --- | --- | --- | --- | --- |
| Graphene/Si  (PVE) | 0.405-1.55 | 1.12 @ 1.55μm | 23.3/23.3 @ 0.532μm | 3.78×10^−9^ @ 1.55μm | 1.32×10^9^ @ 1.55μm | [4] |
| WSe_2_ nano-speckle  (PVE) | 0.532-0.86 | 2.15 @ 0.86μm | 32000/36000 @ 0.86μm | 5.4×10^-11^ @ 0.86μm | 2.5×10^8^ @ 0.86μm | [5] |
| PtSe_2_  (PVE) | 0.375-2 | 0.65 @ 1.55μm | 14.1/15.4 @ 0.808μm | ~8×10^−7^ @ 2μm | ~10^13^ @ 2μm | [6] |
| *a*‑MoSe_2_  (PVE) | 0.405-0.65 | 0.94 @ 0.405μm | 61000/58000 @ 0.405μm | 2.33×10^-11^ @ 0.405μm | 4.29×10^10^ @ 0.405μm | [7] |
| Au-MoS_2_/WO_3_  (PVE) | 0.35-0.95 | 0.00963 @ 0.86μm | 66370/63040 @ 0.86μm | 6.52×10^-9^ @ 0.86μm | 1.029×10^7^ @ 0.86μm | [8] |
| GeSe  (PVE) | 0.22-0.85 | 220 @ 0.85μm | 69000/92000 @ 0.85μm | 9.1×10^-12^ @ 0.85μm | 4.1×10^9^ @ 0.85μm | [9] |
| PtTe_2_/SiNPs  (PVE) | 0.635-11 | 0.48 @ 11μm | 5.1/8.9 @ 0.635μm | 1.76×10^-9^ @ 11μm | 6.4×10^7^ @ 11μm | [10] |
| 2D PtTe_2_ /Si  (PVE) | ~1-7 | 13 @ 2μm | n/a | n/a | 1.21×10^6^ @ 7μm | [11] |
| Bi_2_Te_3_/Sb_2_O_3_/p-Si  (PVE) | 0.365-1.05 | 73.6@0.85μm | 24600/25100 @ 0.85μm | n/a | 7.27×10^10^ @ 0.85μm | [12] |
| Au/2H-MoTe_2_/1T’-MoTe_2_  (PVE) | 0.405-2.2 | 0.0001 @ 2.2μm | 22000/59000 @ 0.66 | n/a | ~10^6^ @ 2.2μm | [13] |
| Ti_3_C_2_T_x_/WS_2_/Si  (PVE) | 0.405-1.65 | 0.00005 @ 1.65μm | 0.5/31.1@0.65μm | n/a | ~8×10^8^ @ 1.65μm | [14] |
| TiO_2_/Pyramid-Si  (PVE) | 0.365-1.305 | 0.057 @ 1.305μm | ˂200000 | n/a | 1.4×10^9^ @ 1.305μm | [15] |
| CdS/Ge  (PVE) | 0.365-1.55 | ~50@1.064μm | 300/245 @ 1.064μm | n/a | 1.4×10^9^ @ 1.066μm | [16] |
| C_60_/SnS_2_  (PVE) | 0.365-0.85 | 700 @ 0.85μm | 39000/40000 @ 0.456μm | n/a | 8.5×10^10^ @ 0.85μm | [17] |
| PdSe_2_/MoTe_2_  (PVE) | 0.3-4.05 | ~0.1 @ 4.05μm | 337/267 @ 0.98μm | n/a | ~1.9×10^11^ @ 0.98μm | [18] |
| PtSe_2_/i-Si/n^+^-Si  (PVE) | 0.532-2.2 | 5.48×10^-5^ @ 1.55μm | 70/290 @ 0.532μm | ~6×10^-13^ @ 0.532μm | 1.94×10^11^@ 0.532μm | [19] |
| SrTiO_3_  (PTE) | 0.325-10.67 | n/a | 1520000/1520000 @ 10.57μm | n/a | n/a | [20] |
| 3D Cd_3_As_2_  (PTE) | 10.4 | 0.17 | 70000/90000 | n/a | n/a | [21] |
| DyTe_3_  (PTE) | 0.38-4.4 | ~20 @ 4.4μm | 78000/106000 @ 4.4μm | n/a | ~7×10^8^ @ 4.4μm | [22] |
| NbTe_4_  (PTE) | 0.532-1.064 | ~0.015 @ 1.064μm | 281000/128000 @ 1.064μm | n/a | n/a | [23] |
| CrSBr  (PTE) | 0.405-1.55 | ~0.1V/W @ 1.55μm | 50/55 @ 0.638μm | n/a | n/a | [24] |
| **(100)-MoO_2_**  **(PTE)** | **0.78-10.5** | **22.4 @ 10.5μm** | **21/30 @ 10.5μm** | **3.08×10^-11^ @ 10.5μm** | **8.59×10^6^ @ 10.5μm** | **This work** |

*Note:* silicon nanopillars (SiNPs); quantum dot (QD); photovoltaic effect (PVE); photothermoelectric effect (PTE).

**Table S2**. Comparable table of the photoresponse performances of broadband self-powered photodetectors on flexible substrate at room temperature.

| **Materials**  **(mechanism)** | **Range**  **(μm)** | ***R_Ph._***  **(mA/W)** | ***t_on_*/*t_off_***  **(ms)** | ***NEP***  **(W/Hz^0.5^)** | ***D****  **(Jones)** | **Ref.** |
| --- | --- | --- | --- | --- | --- | --- |
| HgTe CQDs  (PVE) | 1.5-2.5 & 3-5 | 220 @ 2.2μm | 0.000012/0.00026 @ 2.2μm | 6.7×10^-13^ @ 2.2μm | 7.5×10^10^ @ 2.2μm | [25] |
| 1D Te-NRs  (PTE) | 0.405-4.06 | ~0.5V/W @ 4.06μm | 8.3/8.8 @ 0.52μm | n/a | ~5×10^9^ @ 4.06μm | [26] |
| Graphene/CH_3_NH_3_PbI_3_  (PVE&PTE) | 0.5-0.98 | ~0.00182 @ 0.98μm | ~2500/25000 @ 0.98μm | 1.2110^-9^ @ 0.98μm | 6.9×10^9^ @ 0.98μm | [27] |
| MoSe_2_/FePS_3_  (PVE) | 0.35-0.9 | 52 @ 0.522μm | 600/600 @ 0.6μm | 7×10^-12^ @ 0.9μm | 8×10^9^ @ 0.9μm | [28] |
| Te/Ag_2_Te/Ag NW  (PTE) | 0.365-1.2 | ~2 V/W @ 1.2μm | 450/390 @ 1.2μm | ~2.2×10^-9^ @ 1.2μm | ~3.6×10^8^ @ 1.2μm | [29] |
| ITO/MoS_2_/WSe_2_/ SWCNTs  (PVE) | 0.47-0.84 | ~1.6 @ 0.84μm | 0.46/0.54 @ 0.532μm | n/a | ~2.4×10^9^ @ 0.84μm | [30] |
| GaSe/MoS_2_  (PVE) | 0.375-0.633 | ~1000 @ 0.45μm | 5/5 @ 0.45μm | n/a | ~6.5×10^9^ @ 0.45μm | [31] |
| (FASnI_3_)_0.1_(MAPbI_3_)_0.9_  (PVE) | 0.35-1.0 | ~20 @ 1.0μm | 0.022/0.020 @ 0.808μm | n/a | ~3.3×10^9^ @ 1.0μm | [32] |
| Graphene/Cu_3-x_P  (PVE) | 0.4-1.55 | ~6000 @ 1.55μm | 419/1090 @ 0.405μm | n/a | n/a | [33] |
| n-InSe/p-InSe  (PVE) | 0.98 | 0.5 | 8.3/9.6 | n/a | n/a | [34] |
| MoS_2_/Al_2_O_3_/Si  (PVE) | 0.405-0.98 | 58 @ 0.83μm | 0.0256/0.0032 @ 0.98μm | n/a | 7.83×10^11^ @ 0.83μm | [35] |
| Ag/Si-NWs/Si/[Si-NWs/rGO]/rGO/Au  (PVE) | 0.52-10 | 1.4 @ 1.55μm | 73/142 | n/a | 1.07×10^7^ @ 10μm | [36] |
| Si/[Si-NWs/FASnBr_3_]/MoO_3_/Au  (PVE) | 0.405-0.98 | 241 @ 0.98μm | ~59/44 @ 0.98μm | n/a | ~10^12^ @ 0.98μm | [37] |
| nf-MoS_2_/Si_3_N_4_  (PVE) | 0.95 | 1358 | 0.69/0.71 | 4.5 × 10^−13^ | 2.8×10^10^ | [38] |
| MoO_3_/Al_2_O_3_/n-Si  (PVE) | 0.405-0.98 | 696 @ 0.98μm | 5.33 /0.97μs @ 0.98μm | n/a | 1.58×10^13^ @ 0.98μm | [39] |
| HA_2_EA_2_Pb_3_I_10_  (PVE) | 0.405-0.94 | n/a | 0.3/0.32 | n/a | n/a | [40] |
| PdTe_2_/thin Si  (PVE) | 0.3-1.2 | ~10 @ 1.2μm | 0.0045/0.379 @ 0.73μm | ~10^-13^ @ 0.73μm | ~10^12^ @ 0.73μm | [41] |
| MoS_2_/Se  (PVE) | 0.48-1.1 | 490 @ NIR | 950/2120 @ NIR | n/a | ~10^10^ @ NIR | [42] |
| MoS_2_/Sb_2_Te_3_  (PVE) | 0.5-0.9 | ~0.1 @ 0.9μm | n/a | n/a | n/a | [43] |
| **(100)-MoO_2_**  **(PTE)** | **0.5-10.5** | **107.31 @ 10.5μm** | **0.121/0.158 @ 10.5μm** | **6.64×10^-12^ @ 10.5μm** | **4.0×10^7^ @ 10.5μm** | **This work** |

*Note*: Nanowire (NW); Colloidal quantum dot (CQD); Ethylammonium (EA); n-hexylammonium (HA)

**Reference**

[1] a) J. Jiang, D. Yang, Y. Wang, X. Guo, M. Yassine, W. Huang, T. Xu, S. Wang, H. Huang, F. Ouyang, Y. Gao, *Appl. Surf. Sci.* **2022**, *606*, 154983; b) D. Wu, Y. Yang, P. Zhu, X. Zheng, X. Chen, J. Shi, F. Song, X. Gao, X. Zhang, F. Ouyang, X. Xiong, Y. Gao, H. Huang, *J. Phys. Chem. C* **2018**, *122*, 1860.

[2] a) H. Wu, P. Tong, N. Li, X. Zhou, N. Wei, J. Zhao, *ACS Appl. Nano Mater.* **2022**, *5*, 16633; b) C. Zhang, X. Zou, Z. Du, J. Gu, S. Li, B. Li, S. Yang, *Small* **2018**, *14*, 1703960.

[3] L. Wang, J. Chen, S. J. Cox, L. Liu, G. C. Sosso, N. Li, P. Gao, A. Michaelides, E. Wang, X. Bai, *Phys. Rev. Lett.* **2021**, *126*, 136001.

[4] M. Qasim, M. Sulaman, A. Bukhtiar, B. Deng, A. Jalal, Y. Sandali, N. H. Shah, C. Li, G. Dastgeer, H. Bin, *Energy Technol.* **2023**, *11*, 2300492.

[5] A. Sharma, U. Varshney, A. Yadav, P. Vashishtha, L. Goswami, G. Gupta, *Mater. Res. Bull.* **2024**, *169*, 112518.

[6] P. Ye, H. Xiao, Q. Zhu, Y. Kong, Y. Tang, M. Xu, *Sci. China Mater.* **2023**, *66*, 193.

[7] J. Zhong, X. Zhang, G. Xiang, *ACS Appl. Mater. Interfaces* **2023**, *15*, 56049.

[8] A. Yadav, P. Vashishtha, L. Goswami, P. Kumari, A. Khan, R. Yadav, A. Sharma, P. Prajapat, G. Gupta, *ACS Appl. Opt. Mater.* **2024**, *2*, 784.

[9] M. Hussain, S. Aftab, S. H. A. Jaffery, A. Ali, S. Hussain, D. N. Cong, R. Akhtar, Y. Seo, J. Eom, P. Gautam, H. Noh, J. Jung, *Sci. Rep.* **2020**, *10*, 9374.

[10] Y. Wu, C. Nie, F. Sun, X. Jiang, X. Zhang, J. Fu, Y. Peng, X. Wei, *ACS Appl. Mater. Interfaces* **2024**, *16*, 22632.

[11] M. S. Shawkat, S. B. Hafiz, M. M. Islam, S. A. Mofid, M. M. Al Mahfuz, A. Biswas, H.-S. Chung, E. Okogbue, T.-J. Ko, D. Chanda, T. Roy, D.-K. Ko, Y. Jung, *ACS Appl. Mater. Interfaces* **2021**, *13*, 15542.

[12] W. Wu, W. He, D. Ling, L. Chen, Y. Zhang, X. Fan, Y. Bi, D. Wang, J. Wang, *Small* **2025**, *n/a*, 2501484.

[13] D. Wang, H. Li, J. Liu, Y. Qin, J. Zhao, P. Hou, *ACS Appl. Mater. Interfaces* **2025**, *17*, 33089.

[14] Y. Zhang, C. Cheng, H. Chen, Y. Liu, Q. Li, Y. Gao, Z. Xu, J. Chen, J. Xu, *Adv. Optical Mater.* **2025**, *13*, 2500173.

[15] L. Wu, X. Shi, H. Fan, Q. Li, P. Hu, F. Teng, *Chinese Phys. B* **2025**, <https://doi.org/10.1088/1674>.

[16] X. Luo, J. Li, Z. He, X. Ma, Q. Qin, W. Chen, Z. Xu, Z. Qiu, Y. Wang, L. Li, D. Shi, *Adv. Photon. Res.* **2025**, *6*, 2400190.

[17] M. Kumar, S.-C. Chen, A. Saravanan, B.-R. Huang, H. Sun, *Small* **2025**, *21*, 2411859.

[18] W. Liu, Y. Wu, X. Bao, L. Sun, Y. Xie, Y. Chen, *Adv. Funct. Mater.* **2025**, *n/a*, 2421525.

[19] X. Xu, S. Ke, T. Ji, M. Ge, Z. Li, Y. Chen, B. Liu, Z. Huang, J. Zhou, G. Liu, S. Ke, X. Chen, *ACS Appl. Mater. Interfaces* **2025**, *17*, 15579.

[20] X. Lu, P. Jiang, X. Bao, *Nat. Commun.* **2019**, *10*, 138.

[21] J. Brady, A. Rashidi, S. Ahadi, S. Stemmer, *Appl. Phys. Lett.* **2025**, *126*, 181102.

[22] T. Zhang, Z. Yu, H. Gu, S. Xiong, Y. Chen, Z. Gao, Y. Wang, Y. Yang, F. Sun, T. Zhou, J. Zhang, H. Zhu, Y. Shan, H. Chen, N. Dai, *Adv. Funct. Mater.* **2025**, *35*, 2501917.

[23] H. Luo, F. Wu, C. He, W. Shen, C. Hu, W. Zhao, P. Yu, G. Yang, *NPG Asia Mater.* **2024**, *16*, 61.

[24] J. Zhou, Y. Yang, S. Li, Y. Li, K. Ni, Y. Li, A. Söll, W. Gao, X. Chen, Y. Jiang, L. Li, Y. Yan, C. Hu, W. Shen, Z. Sofer, P. Gong, M. Tian, X. Liu, *ACS Photonics* **2025**, *12*, 2595.

[25] X. Tang, M. M. Ackerman, G. Shen, P. Guyot-Sionnest, *Small* **2019**, *15*, 1804920.

[26] M. Zhang, Y. Liu, F. Guo, B. Zhang, B. Hu, S. Li, W. Yu, L. Hao, *ACS Appl. Mater. Interfaces* **2024**, *16*, 6152.

[27] F. Huang, L. Shen, S. Zhou, S. Wang, S. Wang, G. Deng, S. Zhou, *Opt. Mater.* **2022**, *128*, 112364.

[28] J. Duan, P. Chava, M. Ghorbani-Asl, Y. Lu, D. Erb, L. Hu, A. Echresh, L. Rebohle, A. Erbe, A. V. Krasheninnikov, M. Helm, Y.-J. Zeng, S. Zhou, S. Prucnal, *ACS Appl. Mater. Interfaces* **2022**, *14*, 11927.

[29] R. Wang, Z. He, J.-L. Wang, J.-Y. Liu, J.-W. Liu, S.-H. Yu, *Nano Lett.* **2022**, *22*, 5929.

[30] E.-X. Ding, A. Karakassides, Y. Zhou, R. Fang, F. Ali, E. I. Kauppinen, Z. Sun, H. Lipsanen, *Nano Energy* **2025**, *140*, 111062.

[31] Z. Zou, J. Liang, X. Zhang, C. Ma, P. Xu, X. Yang, Z. Zeng, X. Sun, C. Zhu, D. Liang, X. Zhuang, D. Li, A. Pan, *ACS Nano* **2021**, *15*, 10039.

[32] Z. Chang, Z. Lu, W. Deng, Y. Shi, Y. Sun, X. Zhang, J. Jie, *Nanoscale* **2023**, *15*, 5053.

[33] T. Sun, Y. Wang, W. Yu, Y. Wang, Z. Dai, Z. Liu, B. N. Shivananju, Y. Zhang, K. Fu, B. Shabbir, W. Ma, S. Li, Q. Bao, *Small* **2017**, *13*, 1701881.

[34] C. Patil, C. Dong, H. Wang, B. M. Nouri, S. Krylyuk, H. Zhang, A. V. Davydov, H. Dalir, V. J. Sorger, *Photonics Res.* **2022**, *10*, A97.

[35] Z. Yue, H. Shen, C. Wang, Y. Xu, Y. Li, J. Zheng, J. Chen, H. Li, J. Zeng, L. Wang, *Appl. Surf. Sci.* **2024**, *655*, 159630.

[36] H. Xin, S. Yang, Y. Wang, M. Sulaman, Z. Zhang, Z. Ge, J. Hu, S. Wang, B. Zou, L. Tang, *J. Mater. Chem. C* **2024**, *12*, 3105.

[37] Z. Zhang, S. Yang, Z. Ge, H. Xin, Y. Wang, Y. Jiang, B. Zou, *IEEE Sens. J.* **2024**, *24*, 21792.

[38] P. Vashishtha, P. Prajapat, A. Sharma, P. Goswami, S. Walia, G. Gupta, *Mater. Res. Bull.* **2023**, *164*, 112260.

[39] Y. Xu, H. Shen, Y. Li, Z. Yue, W. Zhang, Q. Zhao, Z. Wang, *ACS Appl. Electron. Mater.* **2022**, *4*, 4641.

[40] S. Han, Y. Ma, L. Hua, L. Tang, B. Wang, Z. Sun, J. Luo, *J. Am. Chem. Soc.* **2022**, *144*, 20315.

[41] C. Dong, X. An, Z. Wu, Z. Zhu, C. Xie, J.-A. Huang, L. Luo, *J. Semicond.* **2023**, *44*, 112001.

[42] D. Dutta, K. S. Reddy, S. Badhulika, *Mater. Sci. Semicond. Process* **2023**, *164*, 107610.

[43] H. Wang, C. Dong, Y. Gui, J. Ye, S. Altaleb, M. Thomaschewski, B. Movahhed Nouri, C. Patil, H. Dalir, V. J. Sorger, *Nanomaterials* **2023**, *13*, 1973.
